# Combined metabolomic and transcriptomic analysis reveals key components of *OsCIPK17* overexpression improves drought tolerance in rice

**DOI:** 10.3389/fpls.2022.1043757

**Published:** 2023-01-09

**Authors:** Shuai Lu, Yaoyu Chen, Surong Wang, Binying Han, Chenglei Zhao, Penghui Xue, Yue Zhang, Hui Fang, Baohua Wang, Yunying Cao

**Affiliations:** School of Life Sciences, Nantong University, Nantong, China

**Keywords:** *Oryza sativa*, CIPK, transcriptome, metabolome, citric acid, auxin, drought

## Abstract

Oryza Sativa is one of the most important food crops in China, which is easily affected by drought during its growth and development. As a member of the calcium signaling pathway, CBL-interacting protein kinase (CIPK) plays an important role in plant growth and development as well as environmental stress. However, there is no report on the function and mechanism of OsCIPK17 in rice drought resistance. We combined transcriptional and metabonomic analysis to clarify the specific mechanism of OsCIPK17 in response to rice drought tolerance. The results showed that OsCIPK17 improved drought resistance of rice by regulating deep roots under drought stress; Response to drought by regulating the energy metabolism pathway and controlling the accumulation of citric acid in the tricarboxylic acid (TCA) cycle; Our exogenous experiments also proved that OsCIPK17 responds to citric acid, and this process involves the auxin metabolism pathway; Exogenous citric acid can improve the drought resistance of overexpression plants. Our research reveals that OsCIPK17 positively regulates rice drought resistance and participates in the accumulation of citric acid in the TCA cycle, providing new insights for rice drought resistance.

## Introduction

Climate change has led to a hotter and drier world, leading to droughts which is disadvantageous for agriculture, humans, and livestock. Drought caused by global warming is one of the most severe threats to agricultural and forestry production worldwide. The annual crop loss caused by drought is greater than that caused by all pathogens combined (Gupta et al., 2020). To adapt to soil water deficit, plants respond to drought stress by modifying their physiological characteristics, root growth and structure, and stomatal conductance in the above ground parts. Such tissue-specific responses alter intracellular signal transduction, leading to early flowering or growth inhibition, which typically reduces yield (Kannenberg et al., 2018). Rice is one of the most important cereal crops and the staple food of over half of the world’s population (Emerick and Ronald, 2019). Therefore, drought tolerance genes in rice must be explored and their drought tolerance mechanisms elucidated.

Calcium ions (Ca^2+^) act as important second messengers regulating plant growth, development, and abiotic stress responses (Song et al., 2008). The calcineurin B-like (CBL) protein is one of the major Ca^2+^ sensors (Kudla et al., 2018). It interacts with the CBL-interacting protein kinase (CIPK) to regulate downstream responses (Tang et al., 2020; Verma et al., 2021). However, CBLs and CIPKs are not one-to-one. For example, there are 10 CBLs and 26 CIPKs in *Arabidopsis* and 10 CBLs and 33 CIPKs in rice, implying that CBLs and CIPKs form complex regulatory networks (Kolukisaoglu et al., 2004; Kanwar et al., 2014; Zhang et al., 2014). Different CBL–CIPK complexes mediate a multitude of responses to environmental stresses, such as salinity, cold, and drought, by regulating downstream gene expression and protein activation (Du et al., 2021). The pivotal role of the SOS pathway in salt tolerance is well-known. In *Arabidopsis*, the SOS3/AtCBL4–SOS2/AtCIPK24 complex catalyzes SOS1 phosphorylation and activation and transfers excess Na^+^ to the soil (Zhu, 2003). In rice, *OsCIPK23* expression is induced by pollination, abiotic stress, and exogenous plant hormone treatment, and *OsCIPK23* downregulation reduces seed-setting rate and drought tolerance (Yang et al., 2008). In apple, MdCIPK13 phosphorylates the sucrose transporter MdSUT2.2 at Ser^254^ to fine-tune salt tolerance (Ma et al., 2019). In potato, StCIPK10 enhances both free radical scavenging ability and the corresponding osmoregulator contents, thereby strengthening drought and osmotic stress tolerance (Ma et al., 2021b). In pepper, CaCIPK3 acts as a positive regulator of drought stress tolerance *via* the CBL–CIPK network to regulate MeJA signaling and the antioxidant defense system (Ma et al., 2021a). In wheat, TaCIPK23 plays important roles by mediating the crosstalk between ABA signaling and drought stress response (Cui et al., 2018). However, current research on the involvement of CIPK in drought stress mainly focused on reducing reactive oxygen species (ROS) and responding to the ABA signaling pathways (Aslam et al., 2022; Li et al., 2022). In addition, it is unclear whether CIPK is involved in trehalose and tricarboxylic acid (TCA) cycle metabolism to maintain intracellular homeostasis.

Trehalose is a disaccharide of glucose. It is a natural, non-toxic, and irreducing bioactive sugar (O'Hara et al., 2013). Many organisms synthesize trehalose upon exposure of cells to stressful conditions, including dehydration, heat, oxidation, hypoxia, and hyperoxia (Lunn et al., 2014). Trehalose promotes wound healing by protecting cells, particularly cell membranes, from oxidative damage and drying (Cejka et al., 2019). In tomato, trehalose enhanced drought tolerance, induced defense, and augmented resistance to bacterial wilt (MacIntyre et al., 2022). Trehalose-6-phosphate synthase (TPS) catalyzes the first step in trehalose-6-phosphate (T6P) and trehalose biosynthesis (Morabito et al., 2021). Apparently, the TCA cycle-associated proteins are upregulated under drought stress (Wang et al., 2019). Previous metabolomic analyses have revealed ectopic expression of the *Prunus* HXK3 gene in *Arabidopsis* increased the contents of phosphorylated sugars, starch, and some TCA cycle-related metabolites to enhance drought tolerance (Pérez-Díaz et al., 2021).

Studies on complete dehydration tolerance in insects revealed that during preparation or recovery from disaster, gene regulation alone cannot explain the rapid biochemical reactions and independent metabolic changes (Ryabova et al., 2020). Therefore, both gene and non-enzymatic chemical cascades may work synergistically to regulate plant drought tolerance. In a previous study, we found *OsCIPK17* overexpression improved rice drought tolerance (Gao et al., 2022a). Therefore, we sequenced the transcriptome and metabolome of the wildtype (WT) Nipponbare (NIP) and *OsCIPK17-*overexpressing lines (OsCIPK17-OE) to identify the key genes and metabolites involved in the regulation of rice drought resistance and clarify the specific regulatory factors.

## Materials and methods

### Construction, transformation and identification of *OsCIPK17*-mutant and *OsCIPK17*-OE transgenic lines

The vector of *OsCIPK17* knockout mutant was constructed by Wuhan Boyuan Biological Company using the CRISPR-Cas9 method. The overexpression vector was constructed by cloning *OsCIPK17* coding sequence into PCM1307 vector and sent to Wuhan Boyuan Biological Company. The successfully constructed vector was introduced into rice embryogenic callus and differentiation culture by Agrobacterium tumefaciens mediated method. After hygromycin screening, the DNA of resistant differentiated plantlets of *OsCIPK17* mutant was extracted, specific primers were designed on both sides of the target, a PCR detection reaction was conducted, and the transformation rate was counted to obtain transgenic rice plantlets *in vitro*. At the same time, T2 generation field seedlings were identified by cloning and sequencing. Homozygous single-copy transgenic lines were used in all experiments. The T0 generation of overexpressed *OsCIPK17* plant*s* were identified by qPCR, and the specific primers for fluorescence quantification were shown in **Table S1**.

### Plant materials and growth conditions

Rice seeds were germinated and grown in vermiculite and nutrient soil (v: v = 1:3) in an artificial climate chamber (16 h light, 8 h darkness, 23 °C). After 10 days of germination, the seedlings were thinned to ensure the same number in each pot. All plants grew under the same water conditions until 3-week-old. Watering was withheld and photographs were obtained after 2 weeks to record the drought phenotype. The whole process of drought-treated rice seedlings was darked-treated, and the rest of the external growth conditions were carried out according to normal light conditions. Under hydroponic culture conditions, after 10 days of germination, the Kimura B nutrient solution was changed every 3 days. When the plant grows to 3 weeks old, 20% PEG6000 (w: v) was used to simulate drought for 5 days, the roots and leaves were collected separately and stored at -80°C for sequencing and physiological measurement. In the condition of hydroponics, 25 μmol L^-1^ citric acid (Tahjib-Ul-Arif et al., 2021) was used for seed soaking, and water was used as control. 14 days later, photographs were recorded and samples were taken. The roots were collected and stored at -80°C for subsequent experiments. In this experiment, physiological indexes, fluorescence quantification and materials of omics were all carried out under the condition of hydroponics.

### RNA-Seq and data analysis

Suzhou PANOMIX Biomedical Tech Co., LTD sequenced the transcriptome of NIP and OE9 roots under normal (NIP-CK and OE9-CK) and drought (NIP-D and OE9-D) conditions. There were four treatments, with three independent replicates per treatment, yielding a total of 12 samples. The original RNA-seq data were submitted to the National Genomics Data Center (Bioproject: PRJCA012202). Quality control was confirmed using the Illumina HiSeq software, and all readings that passed the filter specifications were plotted on the reference genome IRGSP-1.0. Sanger quality value was used to evaluate the sequencing quality of offline data. Quality value, referred to as Q value, is the result of rounded map of the base read error rate p. FASTQ file are encoded using Illumina 1.8+version. The difference between the ASCII value of all characters and the offset value of 33 is denoted as the Q value of the base. For example, if the ASCII value of character I is 73, then the base mass of the corresponding position of the character is 40 (73 minus 33), and the sequencing error rate is 0.01%. Before differential expression analysis, the correlation of gene expression levels among samples was checked. Using the R package DESeq, PCA was performed on each sample according to the expression. After calculating the expression level of each transcript and gene, DESeq was used for differential expression analysis. The conditions for screening DEGs were as follows: expression difference in multiple |log2foldchange| > 1 and significance p value < 0.05. A volcano map of DEGs was drawn using the R package ggplot2. KEGG and GO enrichment analyses were performed using OmicShare (Wang et al., 2021) (www.omicshare.com/tools).

### Metabolomic analysis

Suzhou PANOMIX Biomedical Tech Co., LTD performed non-targeted metabolomics. There were four treatments, with six independent replicates per treatment, yielding a total of 24 samples. The original metabolome data were submitted to the National Genomics Data Center (Bioproject: PRJCA012202). The reagents and methods used for extracting metabolites were carried out as described by Vasilev et al. (2016). Liquid chromatography (LC) was performed using the Vanquish UHPLC System (Thermo Fisher Scientific, USA). Chromatography was performed using ACQUITY UPLC ^®^ HSS T3 (150×2.1 mm, 1.8 µm) (Waters, Milford, MA, USA). The column was maintained at 40°C. The flow rate and injection volume were set at 0.25 mL/min and 2 μL, respectively. For LC-ESI (+)-MS analysis, the mobile phases consisted of 0.1% formic acid in acetonitrile (v/v) and 0.1% formic acid in water (v/v). For LC-ESI (-)-MS analysis, the analytes were carried out with acetonitrile and ammonium formate (5mM) (Zelena et al., 2009). Gradient elution was performed following the method described by Zelena et al. Mass spectrometric (MS) detection of metabolites was performed using Orbitrap Exploris 120 with an ESI ion source (Thermo Fisher Scientific, USA). The parameters for positive and negative ion modes were determined as described by Want et al. (2013). The raw data were firstly converted to mzXML format by MSConvert in ProteoWizard software package (v3.0.8789) (Smith et al., 2006) and processed using XCMS (Navarro-Reig et al., 2015) for feature detection, retention time correction and alignment. In metabonomic research based on mass spectrometry, quality control (QC) is required to obtain reliable and high-quality metabonomic data. In this experiment, QC samples are used for quality control during LC-MS detection. To find biomarkers, the relative standard deviation (RSD) of the potential characteristic peaks in the QC samples, that is, the coefficient of variation should not exceed 30%, and the unqualified characteristic peaks should be deleted. Therefore, on the basis of quality control, quality assurance (QA) is usually carried out to delete the features with poor repeatability in QC samples, so as to obtain a higher quality data set, which is more conducive to the detection of biomarkers (Dunn et al., 2011). The metabolites were identified by accuracy mass (< 30 ppm) and MS/MS data which were matched with HMDB (Wishart et al., 2007) (http://www.hmdb.ca), massbank (Horai et al., 2010) (http://www.massbank.jp/), LipidMaps (Sud et al., 2007) (http://www.lipidmaps.org), mzclound (Abdelrazig et al., 2020) (https://www.mzcloud.org) and KEGG (Ogata et al., 1999) (http://www.genome.jp/kegg/). The robust LOESS signal correction (QC-RLSC) (Gagnebin et al., 2017) was applied for data normalization to correct for any systematic bias. After normalization, only ion peaks with relative standard deviations (RSDs) less than 30% in QC were kept to ensure proper metabolite identification. OPLS-DA allowed the determination of discriminating metabolites using the variable importance on projection (VIP). The P value, VIP produced by OPLS-DA, fold change (FC) was applied to discover the contributable-variable for classification. Finally, P value < 0.05 and VIP values > 1 were considered to be statistically significant metabolites. Differential metabolites were subjected to pathway analysis by MetaboAnalyst (Xia and Wishart, 2011), which combines results from powerful pathway enrichment analysis with the pathway topology analysis. The identified metabolites in metabolomics were then mapped to the KEGG pathway for biological interpretation of higher-level systemic functions. The metabolites and corresponding pathways were visualized using KEGG Mapper tool. Transcriptome and metabolome association, O2PLS, and correlation network analyses were performed using OmicShare.

### qRT-PCR analyses of gene expression

Plant RNA extraction was performed according to the instructions of the RNA simple Total RNA Kit from TIANGEN. cDNA synthesis was performed according to the instructions of the FastKing one-step genomic cDNA first strand synthesis premix reagent from TIANGEN. Fluorescent quantitative PCR was performed using the previously synthesized cDNA as a template by following the instructions provided with the FS Universal SYBR Green Master Kit from ROCHE. *OsACTIN-1* (*LOC_Os05g36290*) was used as the internal reference to detect the expression level of each target gene. Specific primers were shown in **Table S1**. The expression of gene is expressed by the relative expression levels (Cao et al., 2013), three or more replicates were set for each sample and the internal reference.

### Y2H assays

For yeast two hybrid (Y2H) assays, the coding and truncated sequences of *OsCIPK17* were obtained by PCR with different pairs of gene specific primers (**Table S1**) and cloned into pGBKT7 vector and pGADT7 vectors. The coding sequence of transcription factors [OsDRO1, OsRACR5, OsNAC5, and OsDREB2B (LOC_Os05g27930)] predicted to interact with OsCIPK17 were connected to the pGADT7 vector and those of other interacting proteins to the pGBKT7 vector. The recombinant pGBKT7 and pGADT7 vectors were transformed into the yeast strain Y2H as the control. The transformed yeast cells were grown on a synthetic dropout minimal medium [(SD)/- Leu/-Trp] and cultured at 30°C for 3 days. A single colony of yeast grown in the previous step was selected, transferred to the SD/-adenine (Ade)/-His/-Leu/-Trp medium, and cultured at 30°C for 3 days. The interaction between OsCIPK17 and the target protein was evaluated according to the growth status on the SD/-Ade/-His/-Leu/-Trp medium and growth status of the negative and positive controls.

### Physiological measurements

RWC was determined according to the previously reported method (Yuan et al., 2019). In brief, the fully expanded leaves of 3-week-old *OsCIPK17*-OE, *OsCIPK17*-Mutant and WT plants were separated to record leaf fresh weight (WF), turgid leaf weight (WT) and dry weight (WD). RWC were calculated according to the following equation: RWC (%) = (WF − WD)/(WT − WD) × 100%. For the measurement of root dry weight, 5-week-old plants were washed and their roots were separated from the soil, dried at 80°C for 3 days, and weighed to determine dry weight.

The soluble sugar content was determined using the anthrone method (Morris, 1948). Briefly, the leaves of 3-week-old plants treated with 20% PEG6000 for 5 days were collected. Leaf tissues (0.5 g) were extracted in 15 mL of distilled water by boiling for 20 min with constant stirring. The supernatant was filtered and raised to a final volume of 100 mL with distilled water. Then, 1 mL of the extract was incubated with 5 mL of anthrone reagent at 95°C for 15 min. The reaction was terminated on ice, followed by the measurement of the absorbance at 620 nm. ROS content was measured using the fluorescent probe H2DCFD (medchemexpress, USA) method in accordance with the manufacturer’s instructions. Pro content and SOD activity were analyzed using commercial kits (Nanjing Jiancheng Bioengineering Institute, Nanjing, China). Contents were detected with a microplate detector (EnSpire (TM) 2300 in the United States) and an ultraviolet spectrophotometer (Evolution 300 in the United States).

### DAB and NBT staining

*In situ* detection of H_2_O_2_ and superoxide anions in leaf tissues was performed using DAB and NBT staining. Leaves of plants exhibiting satisfactory growth under normal and drought conditions were cut into small segments of approximately 2 cm, and 10 segments were placed in the culture plate. Then, 5 mL of staining solution (1 mg·mL^-1^ DAB or 0.5 mg·mL^-1^ NBT) was added, and the samples were incubated in the dark for 12 h. Thereafter, the samples were decolorized in 90% ethanol in a boiling water bath and photographed.

Identification of UGT gene family members and construction of a phylogenetic tree

Ensembl Plants downloads the genome-wide protein and Pfam database UGT gene family HMM file PF00201. UGT family genes were searched using Tbtools (Chen et al., 2020) hmmsearch with default parameters and a threshold E-value < 0.01. Candidate UGT family genes were validated using PFAM and SMART to verify the presence of conserved structural domains. Multiple sequence alignment of the above obtained family members was performed using MUSCLE v.3.8.31 (Edgar, 2004) for the proteins. The sequences were then trimmed with TrimAL v.1.2 (Capella-Gutiérrez et al, 2009). Using IQ-TREE v.2.0 (Nguyen et al., 2015), the best models for UGT were automatically obtained as WAG+F+G4, respectively, according to the Bayesian Information Criterion (BIC). Create profiles using the default settings of PhyloSuite v.1.2.2 and build Bayesian inference trees using MrBayes v.3.2.7a. Phylogenetic trees were edited and annotated with iTOL v.3.

### Statistical analysis

SPSS v.25.0 and GraphPad Prism v.9.0.0 were used for statistical analysis and charting. Significant differences in the figures follows the following rules: rules: ****P<0.0001, ***p < 0.001, **p < 0.01, and *p < 0.05.

## Results

### Generation of *OsCIPK17*-mutant and *OsCIPK17*-OE transgenic lines

To study the function of OsCIPK17, japonica variety Nipponbare was used as the parent to construct mutant (*K17-41* and *K17-44*) and overexpressed lines (OE-8 and OE-9) of *OsCIPK17*. PCR sequencing results showed that the *K17-41* lost two bases “GG” on target 1 of the first exon, added base “A” on the second target, *K17-44* lost one base “G” on target 1 of the first exon, and added base “T” on the second target. qRT-PCR showed that the transcription level of *OsCIPK17* in OE8 and OE9 lines was 2.6 and 4.9 times higher than that in WT (**Figure S1**).

### *OsCIPK17* overexpression enhanced drought tolerance in rice

**Figure 1:**
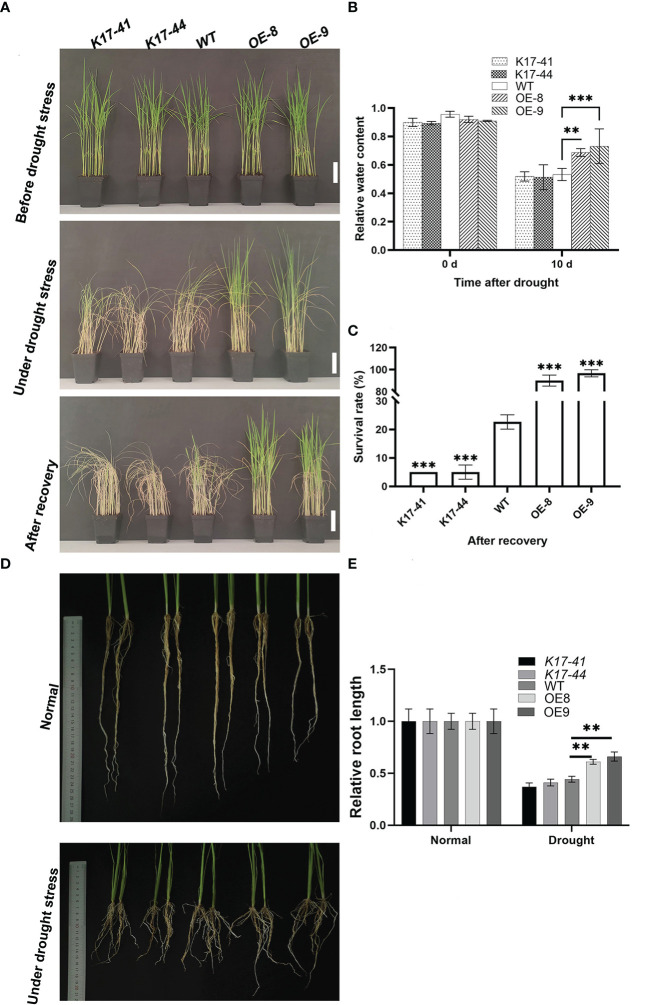
Drought tolerance was increased in *OsCIPK17*-OE plants but decreased in *OsCIPK17*-Mutant plants. **(A)** Growth phenotype of 3-week-old transgenic *OsCIPK17*-OE, *OsCIPK17*-Mutant, and WT plants at different stages of drought stress; bar = 7cm. The upper and middle channels in a have the same results as Gao et al. (2022a) (Published in the International Journal of Molecular Science on 18 October 2022). **(B)** Relative water content of drought-treated plants. Parameters of water status in 3-week-old *OsCIPK17*-OE, *OsCIPK17*-Mutant and WT plants were measured at 10 days after drought stress treatment (n=20). **(C)** Survival rates of each line after recovery were measured at 2 days after re-watering (n=60). **(D)** Growth phenotypes of 5-week-old transgenic *OsCIPK17*-OE, *OsCIPK17*-Mutant and WT plants under drought stress and normal water conditions. **(E)** Root length of each line in **(D)** was measured (n=6). Experiments in **(A, D)** were repeated three times, and similar results were obtained. Data in **(B, C, E)**, are presented as mean ± SD of three independent experiments, and the significant differences between *OsCIPK17*-OE/*OsCIPK17*-Mutant and WT plants according to one-way ANOVA are indicated by asterisks. Asterisks indicate significance (**p < 0.01, ***p < 0.001).

To explore the role of *OsCIPK17* in drought tolerance, seedlings of WT, two *OsCIPK17-OE* (OE8 and OE9) lines, and two *OsCIPK17*-Mutant (K17-41 and K17-44) lines were treated with 20% PEG 6000 for simulating drought stress. Under normal conditions, no noticeable differences were observed among WT, *OsCIPK17*-OE, and *OsCIPK17*-Mutant plants (**Figure 1A**, upper pannel). The seedlings were grown without watering for 14 days after drought treatment. Compared with WT plants, *OsCIPK17*-Mutant plants showed earlier and more severe drought symptoms with rolled leaves and withered plants, whereas *OsCIPK17*-OE plants showed relatively later and milder drought symptoms (**Figure 1A**, middle pannel). This is consistent with our previous study (Gao et al., 2022a). Two days after re-watering, *OsCIPK17*-OE plants showed a higher survival rate, while almost all *OsCIPK17*-Mutant plants died (**Figure 1A**, lower panel). The survival rates of OE8 and OE9 were 83% and 87%, respectively. The survival rate of WT was 25%, whereas that of *K17*-41 and *K17*-44 was 5% (**Figure 1C**). These results showed that *OsCIPK17*-OE improved the drought tolerance of transgenic rice, while *OsCIPK17*-Mutant plants were drought-sensitive. To further confirm the role of *OsCIPK17* in drought tolerance, relative water content (RWC) after drought stress treatment was compared among *OsCIPK17*-OE, *OsCIPK17*-Mutant, and WT plants. The RWC of *OsCIPK17*-OE plants was significantly increased (by 12–17%) compared with that of WT plants (**Figure 1B**), while the RWC of *OsCIPK17*-Mutant plant was comparable to that of WT plants 10 days after treatment. These results confirmed that *OsCIPK17* plays important roles in drought stress tolerance in rice.

### *OsCIPK17* overexpression increased root length under drought

A wider root growth angle and greater root length density help rice plants use limited water to tolerate drought (Uga et al., 2013). The growth of *OsCIPK17*-OE, *OsCIPK17*-Mutant, and WT roots was recorded under normal (**Figure 1D**, upper panel) and drought conditions (**Figure 1D**, lower panel). Under drought conditions, root length of OE plant increased compared to WT and mutant, and the statistics showed that root length of OE plant was 0.5-0.6 under normal conditions, while WT and mutant were 0.3-0.4 under normal conditions. (**Figure 1E**). Deep rooting may help plants to avoid drought-induced stress by taking in water from deep soil layers (Thao and Tran, 2016). To improve the drought tolerance in rice, therefore, the introduction of the deep-rooted traits into shallow-rooted cultivars is considered one of the most promising breeding strategies (Lou et al., 2017). Interestingly, the RWC and root length performance of the mutants was similar to that of WT, suggesting that OsCIPK17 confers drought resistance by increasing root length.

### Altered *OsCIPK17* expression affected the accumulation of stress-related metabolites during drought stress response

**Figure 2:**
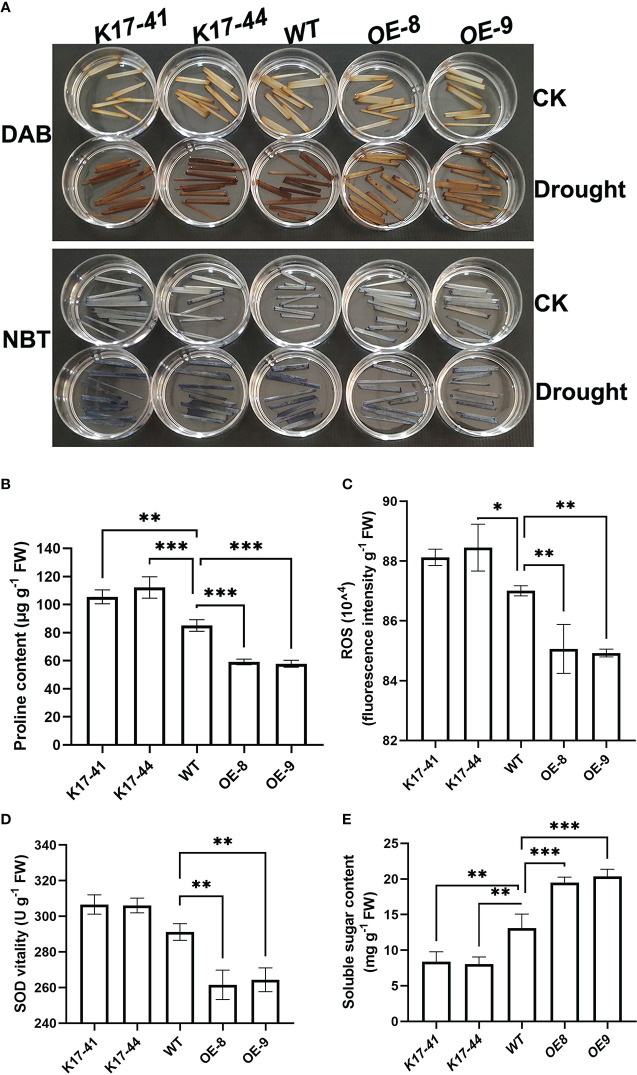
Drought-induced changes in proline, soluble sugar, and ROS levels and SOD activity in *OsCIPK17*-OE and *OsCIPK17*-Mutant plants. **(A)** *In situ* detection of H_2_O_2_ and superoxide anion in leaves using DAB and NBT staining, respectively. **(B)** Proline content, **(C)** ROS content, **(D)** SOD activity. **(E)** Soluble sugar content. Leaf samples were collected from 3-week-old *OsCIPK17*-OE, *OsCIPK17*-Mutant, and WT plants grown under normal water or drought conditions (at 5 days after water withholding) and subjected to physiological measurements. Data in **(B–E)** are presented as mean ± SD of three independent experiments, and significant differences between *OsCIPK17*-OE/*OsCIPK17*-Mutant and WT plants according to one-way ANOVA are indicated by asterisks. Asterisks indicate significance (*p < 0.05, **p < 0.01, ***p < 0.001).

ROS causes oxidative damage. Therefore, ROS accumulation in *OsCIPK17*-OE, *OsCIPK17*-Mutant, and WT plants under drought (20% PEG6000) and normal (normal watering) conditions was analyzed to explore the role of ROS in *OsCIPK17*-mediated drought stress response. Quantitative staining with 3,3 ‘- diaminobenzidine (DAB) (**Figure 2A**, upper panel) and nitrotetrazolium blue (NBT) (**Figure 2A**, lower panel) revealed that 5 days after drought treatment, the staining of H_2_O_2_ and superoxide anions was less obvious in the leaf tissues of *OsCIPK17*-OE plants but more obvious in the leaf tissues of *OsCIPK17*-Mutant plants compared with that in the leaf tissues of WT plants. These observations are consistent with the quantitative results for ROS (**Figure 2C**). To explore the possible physiological basis of *OsCIPK17*-mediated stress response, changes in the levels of some stress-related metabolites, such as proline (Pro) and soluble sugars, as well as in the activity of superoxide dismutase (SOD) were compared among *OsCIPK17*-OE, *OsCIPK17*-Mutant, and WT plants under normal and drought conditions. *OsCIPK17*-OE plants showed a lower Pro content (reduced by 25–30%), while *OsCIPK17*-Mutant plants showed a higher Pro content (increased by 18–23%) than WT plants (**Figure 2B**). Moreover, *OsCIPK17*-OE plants showed a lower SOD activity (decreased by 11–12%) than WT plant; however, there was no difference in SOD activity between OsCIPK17-Mutant and WT plants (**Figure 2D**). In addition, *OsCIPK17*-OE plants showed higher soluble sugar content (enhanced by 58–67%), while *OsCIPK17*-Mutant plants showed lower soluble sugar content (reduced by 30–36%) than WT plants (**Figure 2E**). These results indicate that *OsCIPK17*-OE accumulated less ROS and Pro and presented lower SOD activity. However, soluble sugar content was increased in *OsCIPK17*-OE plants. These results suggest that OsCIPK17-OE has better resistance indicators to combat drought.

### Identification of key genes and metabolites involved in the response of *OsCIPK17*-OE plants to drought

**Figure 3:**
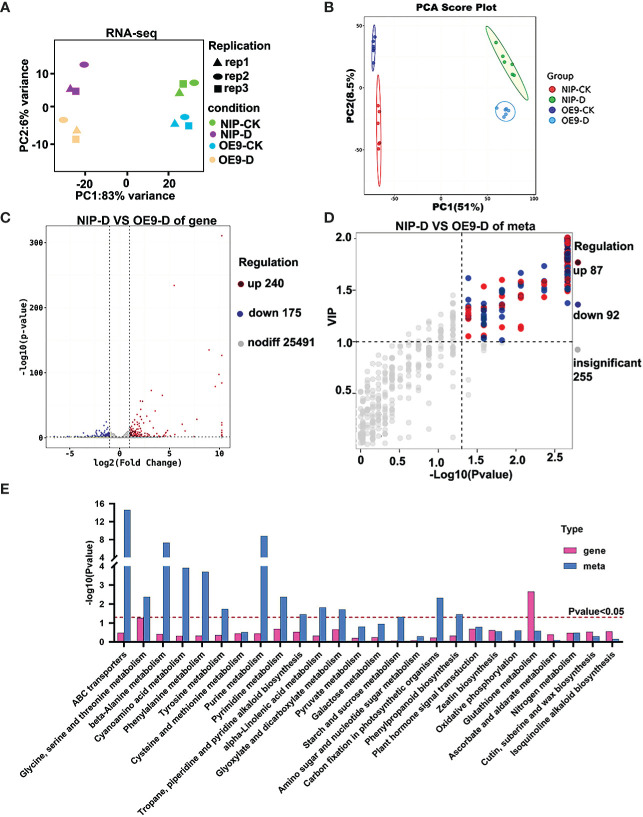
PC biplot of the transcriptome and metabolome of rice seedlings under drought stress and identification of differentially expressed genes and metabolites. **(A)** PC biplot of transcriptome. **(B)** PC biplot of metabolome. **(C)** Volcano plot of differentially expressed genes between NIP and *OsCIPK17*-OE9 under drought treatment. **(D)** Volcano plot of differentially expressed metabolites between NIP and *OsCIPK17*-OE9 under drought treatment. **(E)** KEGG analysis of differentially expressed genes and metabolites between NIP and *OsCIPK17*-OE9 under drought treatment. CK means control check, which means growth under normal conditions without drought treatment. D means under drought treatment. OE9 means *OsCIPK17* overexpression line 9. NIP-D, NIP-CK, OE9-D, and OE9-CK mean combination of lines and treatment methods. The same meaning as the following figure.

To fully understand the transcriptional and metabolic changes in *OsCIPK17*-overexpressing lines, untargeted metabolomic and RNA-Seq analyses were performed under normal growth (control groups, NIP-CK and OE9-CK) and drought treatments (treatment groups, NIP-D and OE9-D) in parent NIP and the *OsCIPK17*-overexpressing line OE-9. Through the quality control of RNA-Seq, the correlation coefficient of gene expression level among all samples was close to 1, showing a high correlation, indicating that the data was reliable (**Figure S7**). The quality control and quality assurance analysis of metabolome showed that QC samples in PCA diagram were clustered, indicating good repeatability and high quality of our data (**Figure S8**). Principal component analysis (PCA) was performed on the transcriptome and metabolome. In RNA-Seq, the control and treatment groups were close to one another, indicating good repeatability and differences (**Figure 3A**). Similar results were observed in the metabolomic analysis (**Figure 3B**). We determined the number of differentially expressed genes (DEGs) and metabolites (DEMs). A total of 25,986 genes were detected in the control groups (NIP-CK *vs* OE9-CK), including 537 DEGs, of which 339 were upregulated and 198 were downregulated (**Figure S2A**). A total of 25,906 genes were detected in the treatment groups (NIP-D *vs* OE9-D), including 415 DEGs, of which 240 were upregulated and 175 were downregulated (**Figure 3C**). Orthogonal partial least squares discriminant analysis (OPLS-DA) was performed on metabolites in positive and negative ion modes (**Figures S3A, B**). A total of 434 metabolites were detected in the control groups, including 176 DEMs, of which 135 were upregulated and 41 were downregulated (**Figure S2B**). A total of 434 metabolites were detected in the treatment groups, including 179 DEMs, of which 87 were upregulated and 92 were downregulated (**Figure 3D**). Venn diagram statistics of total DEGs and DEMs are presented in **Figures S2C, D**. We then classified and counted DEMs in the treatment groups (**Figure S2E**). The major metabolites were amino acids and their derivatives, organic acids, fatty acids, flavonoids and their derivatives, and sugars. Genes detected in this experiment and the metabolic pathways they were involved in is summarized in **Table S1**. Next, the Kyoto Encyclopedia of Genes and Genomes (KEGG) pathways of DEGs and DEMs in the treatment groups were analyzed. KEGG pathway analysis in the control groups showed that the detected DEGs and DEMs were mainly enriched in amino acid metabolism, energy metabolism, nucleotide metabolism, and other items under normal conditions (**Figure S3C, D**). KEGG pathway analysis in the treatment groups showed that the detected DEMs were mainly enriched in amino acid metabolism, energy metabolism, nucleotide metabolism, and other secondary metabolites under drought conditions (**Figure S3F**). These include metabolic items involved in stress response, such as the TCA cycle (Yuenyong et al., 2018), glutathione pathway (Park et al., 2021) and phenylpropane pathway (Zeng et al., 2022). In addition, the detected DEGs were enriched in ascorbic acid (AsA) and sulfur metabolism (**Figure S3E**). KEGG pathway analyses in the treatment group revealed that the detected genes and metabolites were mainly enriched in fundamental metabolic pathways, such as amino acid, nucleotide, and glucose metabolism, in addition to nitrogen metabolism; isoquinoline alkaloid biosynthesis; plant hormone signal transduction; tropine, piperidine, and pyridine alkaloid biosynthesis; phenylpropane biosynthesis; and zeatin biosynthesis (**Figure 3E**). Based on these results, *OsCIPK17* may be involved in these pathways in response to drought.

### *OsCIPK17*-OE increased reducing sugar levels to tolerate drought

**Figure 4:**
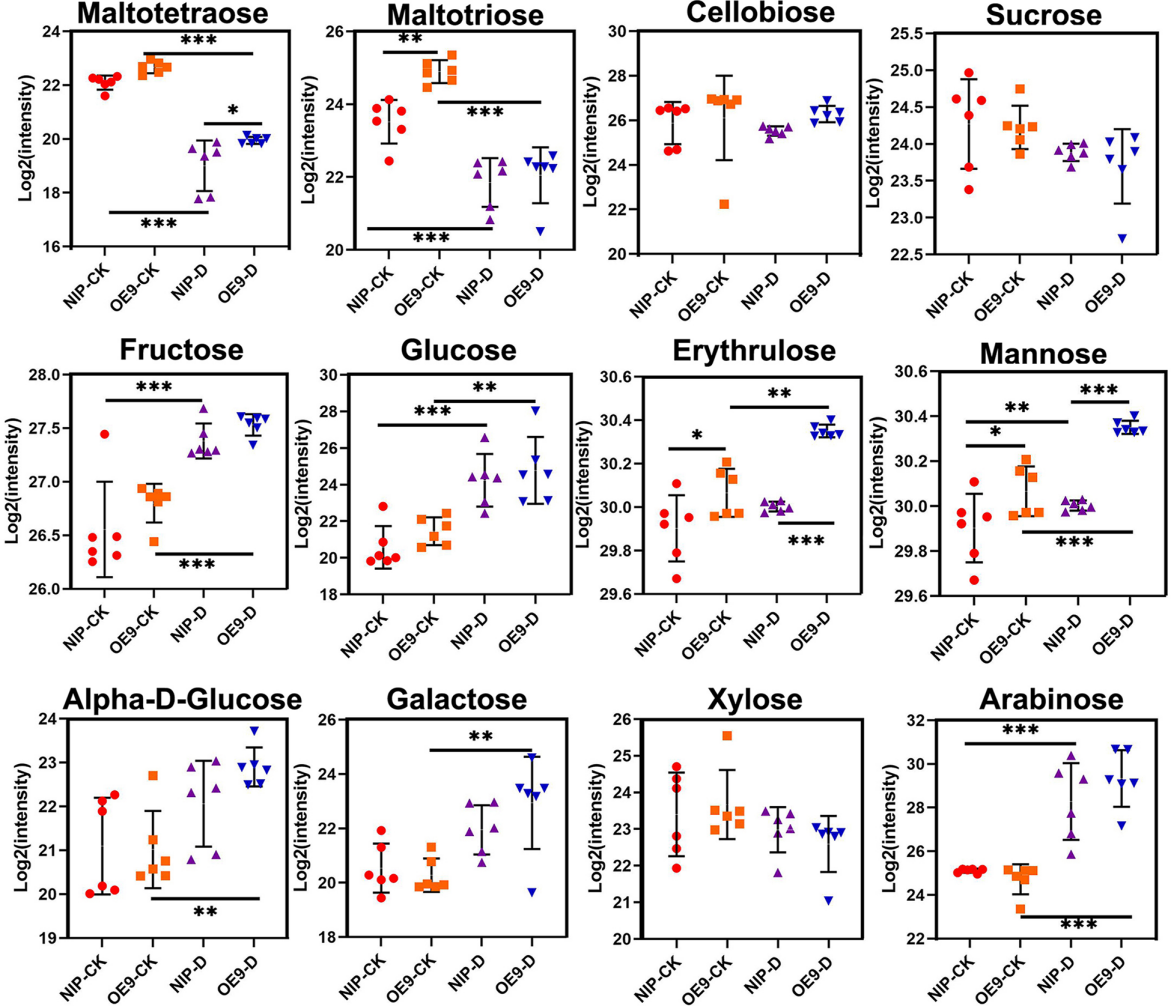
Sugar content in the control and drought treatment with metabolomics analysis. Data are presented as mean ± SD of six independent experiments, and significant differences between NIP-CK and OE9-CK/NIP-D or OE9-D and OE9-CK/NIP-D plants according to one-way ANOVA are indicated by asterisks. Asterisks indicate significance (*p < 0.05, **p < 0.01, ***p < 0.001). CK: NIP or OE materials without drought treatment. OE9: *OsCIPK17*-OE9. D: drought treatment (with 20% PEG treatment of 3-week-old rice seedlings for 5 days).

The leaves of *OsCIPK17-*overexpressing plants accumulated more soluble sugars to tolerate drought (**Figure 2E**). However, the range of soluble sugars was relatively large. To explore the types of sugar playing important roles in drought, the sugar metabolome was analyzed quantitatively. Disaccharides and polysaccharides exhibited a downward trend in both NIP and OE9 under drought treatment (**Figure 4**). For instance, the levels of maltotetraose and maltotriose were significantly decreased in both NIP and OE9 after drought treatment compared with values under normal conditions. Moreover, in the treatment groups, maltotetraose level was significantly higher in OE9 than in NIP, whereas the change in maltotriose level was identical between NIP and OE9 under normal conditions. Cellobiose, sucrose, and xylose levels slightly decreased after drought treatment, albeit without significance. Compared with values in the control groups, fructose, glucose, and arabinose levels significantly increased in the treatment groups. Further, drought stress significantly increased the levels of erythrulose and mannose in OE9 but showed no obvious effect in NIP. Under both normal and drought conditions, erythrulose and mannose levels in OE9 were significantly higher than those in NIP. Drought treatment significantly increased α-D-glucose and galactose levels in OE9 but produced no difference in NIP. Plants prevent the accumulation of toxic metabolites by producing inert counterparts or potential antioxidant compounds (Olszowy, 2019). There was a significant and positive correlation between the total phenol content, reducing sugar content, and antioxidant activity (Khatri and Chhetri, 2020). Our results indicate that reducing sugars, particularly erythrulose, mannose, α-D-glucose, and galactose, may be the key to drought tolerance, playing pivotal roles in the response of *OsCIPK17* to drought.

### *OsCIPK17* participated in trehalose synthesis pathway in response to drought

**Figure 5:**
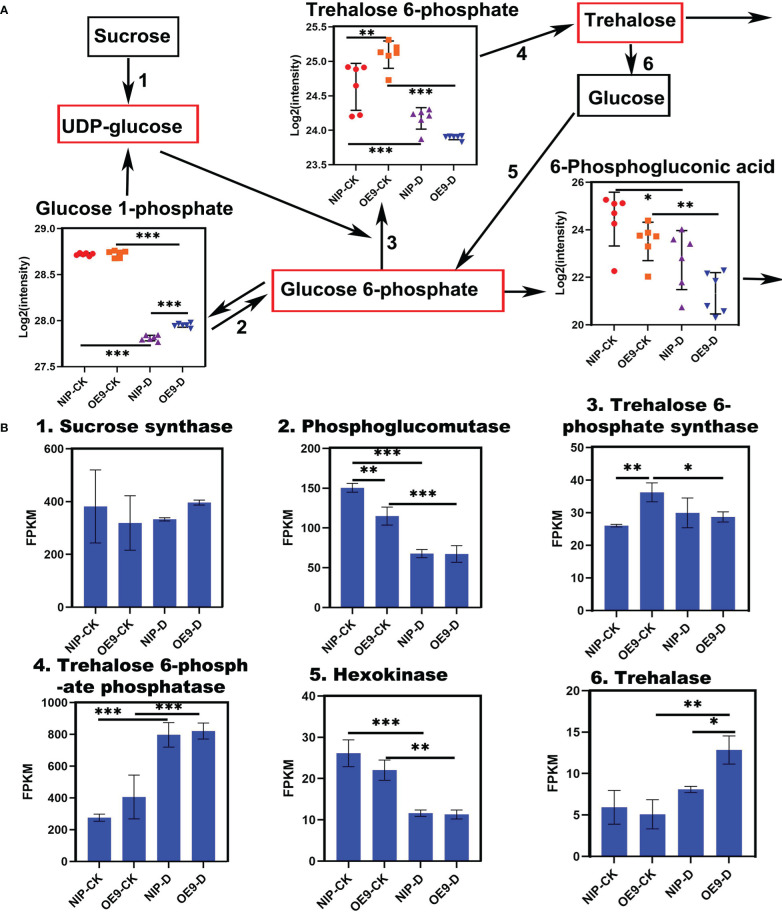
Metabolites and enzymes related to trehalose metabolism. **(A)** Contents of metabolites in the trehalose pathway. Black boxes indicate the substances detected by the metabolomic analysis, and red boxes indicate the substances not detected by the metabolomic analysis. **(B)** Expression of enzymes involved in trehalose pathway detected in the transcriptome. Data are presented as mean ± SD of six independent experiments, and significant differences between NIP-CK and OE9-CK/NIP-D or OE9-D and OE9-CK/NIP-D plants according to one-way ANOVA are indicated by asterisks. CK: NIP or OE9 materials without drought treatment. OE9: *OsCIPK17*-OE9. D: drought treatment. Asterisks indicate significance (*p < 0.05, **p < 0.01, ***p < 0.001).

Screening of DEMs in the treatment group revealed a very interesting substance, T6P. It is an intermediate in trehalose synthesis and is a sugar signal of great significance. It is an important part of mechanism coordinating metabolism with plant growth, adaptation, and development (Paul, 2007). However, trehalose was not included among the quantified metabolites. We mapped the trehalose biosynthetic pathway of T6P (**Figure 5**). TPS catalyzes the transfer of glucose from UDP-glucose to glucose 6-phosphate, forming T6P and UDP. Trehalose phosphate phosphatase (TPP) dephosphorylates T6P to trehalose and inorganic phosphates (**Figures 5A, B**). In the control groups, the T6P level in OE9 was significantly higher than that in NIP. At the early stage of trehalose synthesis, cells accumulate high levels of T6P and UDP-glucose, while the level of T6P decreases following trehalose synthesis (Paul, 2007). This finding is consistent with our results. Specifically, NIP and OE9 were at the late stages of trehalose synthesis following drought treatment. Visual analysis of enzymes involved in the trehalose synthesis pathway showed that the related representative genes exhibited the same change trends. For instance, sucrose synthase, hexokinase and phosphoglucomutase decreased under drought conditions. Under normal conditions, TPS level in OE9 was significantly higher than that in NIP. Following drought treatment, however, the level in OE9 decreased significantly, while that in NIP changed little. TPP level was significantly increased under drought conditions, although there was no difference under normal conditions. In NIP, trehalase level did not differ between normal and drought conditions. However, in OE9, trehalase level significantly increased under drought stress, being higher in OE9-D than in NIP-D. Expression analysis of genes corresponding to the differential metabolite T6P and the whole pathway of its conversion to trehalose revealed that *OsCIPK17* is involved in trehalose synthesis, an important metabolic pathway in response to drought.

### *OsCIPK17* regulated the TCA cycle

**Figure 6:**
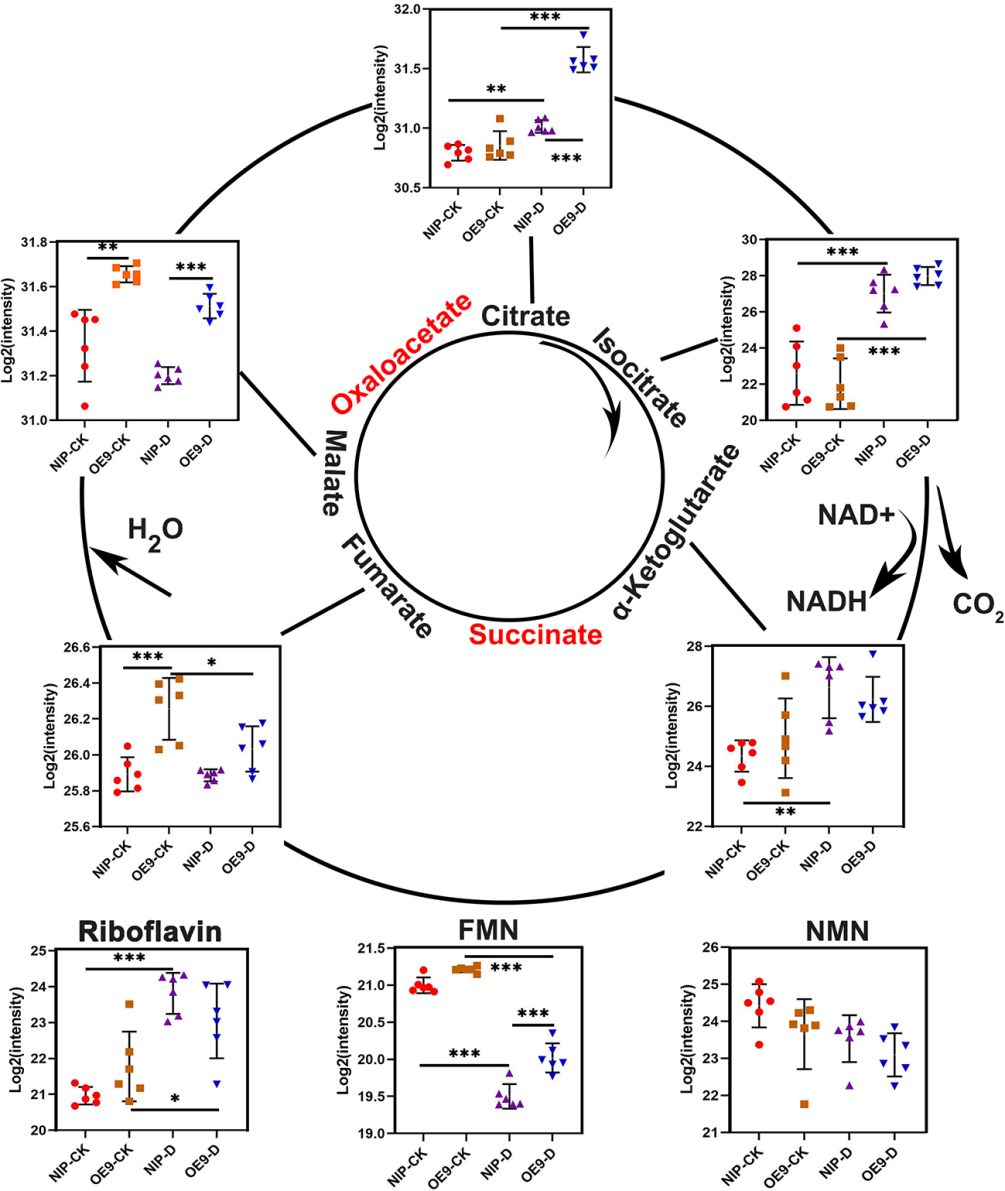
Changes in the contents of intermediate metabolites in the TCA cycle in rice roots under drought. Asterisks indicate significance (*p < 0.05, **p < 0.01, ***p < 0.001).

The TCA or citric acid cycle is a common metabolic pathway in all aerobic organisms and produces energy in the form of adenosine triphosphate (ATP) through a series of cyclic enzymatic reactions (Locasale and Cantley, 2011; Sawa et al., 2017). The first product of the TCA cycle is citric acid, which is the central link between many metabolic pathways and controls cellular energy levels (Arnold et al., 2022). When in excess, citric acid inhibits glycolysis, stimulates gluconeogenesis, and hinders downstream TCA cycle response (Lunt and Vander Heiden, 2011; Lunn et al, 2014). In this study, substances in the TCA cycle showed a general increasing trend under drought conditions, whereas fumarate, and malate showed a decreasing trend. Compared with NIP, OE9 in the treatment group showed a significant increase in citric acid and malate (**Figure 6**). Citric acid accumulation plays an important role in drought response. Citric acid exists in anhydrous form and acts as a chelating, antioxidant, and anticoagulative agent (Ryan et al., 2019). During rehydration, citric acid reserve in the mitochondria may allow a relatively rapid restart of TCA function and energy metabolism without glycolytic carbohydrate input in the cytoplasm (Martínez-Reyes and Chandel, 2020). Increased malate and citrate levels may be involved in the glutamine decomposition pathway and glucose-independent TCA cycle, while glutamine metabolism plays an important role in cell survival and proliferation under hypoxia and glucose deficiency (Le et al., 2012). Collectively, from the previous and present results, transformation of components of the TCA cycle indicates that *OsCIPK17*-overexpressing plants alter cellular energy metabolism by increasing citric acid and malic acid levels to maintain optimal growth and survive until drought ends.

### Under exogenous citric acid treatment, OE plants increased in height and root length and drought tolerance

**Figure 7:**
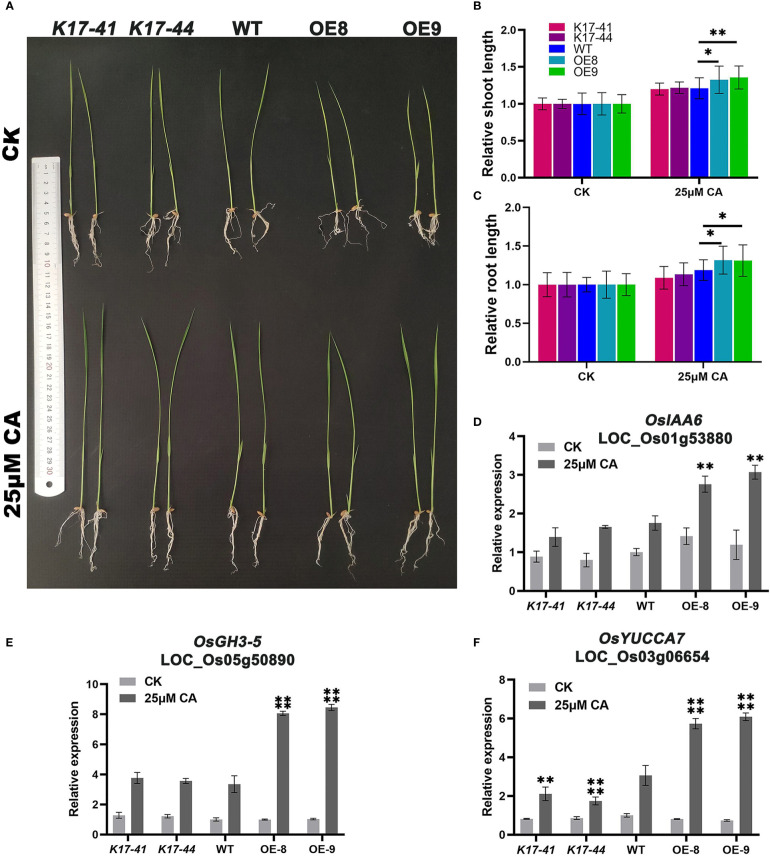
Exogenous citric acid stimulates the growth of *OsCIPK17*-OE plants. **(A)** Growth performance of *OsCIPK17*-OE, *OsCIPK17*-Mutant, and WT seedlings grown in clear water with or without 25 μmol·L^-1^. **(B)** Relative shoot length and **(C)** relative root length of *OsCIPK17*-OE, *OsCIPK17*-Mutant, and WT seedlings grown in clear water with or without 25 μmol·L^-1^ at 14 days after germination. **(D–F)** are the expression levels of citric acid treated and controlled with auxin-related genes in **(A)**. Relative expression levels of the gene were normalized to the internal control of the *OsACTIN-1* (*LOC_Os05g36290*) gene. Data in **(B–F)** are presented as mean ± SD of three independent experiments, and significant differences between *sCIPK17*-OE/*OsCIPK17*-Mutant and WT plants according to one-way ANOVA are indicated by asterisks. Asterisks indicate significance (*p < 0.05, **p < 0.01, ****P<0.0001).

**Figure 8:**
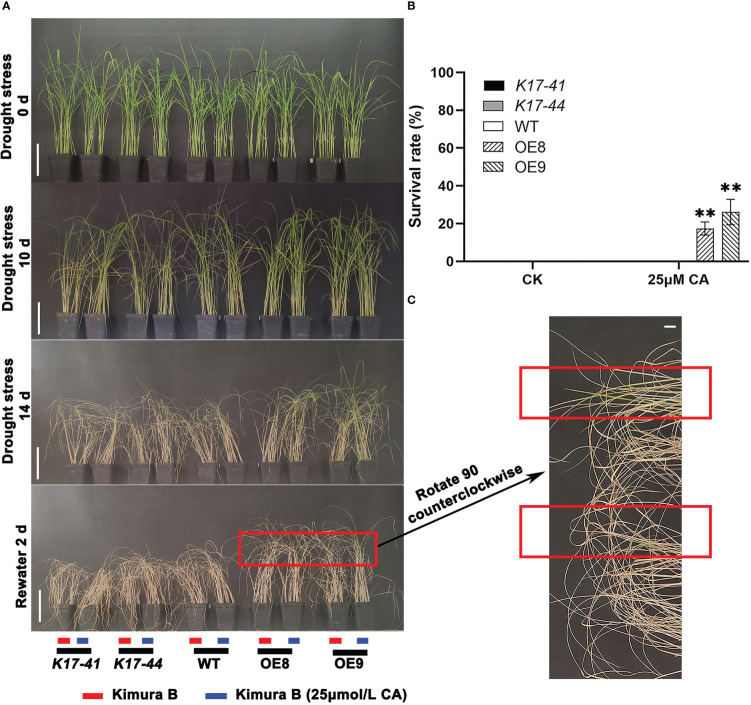
Exogenous citric acid treatment enhanced the drought tolerance of *OsCIPK17*-OE, while it did not affect *OsCIPK17*-Mutant and WT. **(A)** Twenty-two days after germination, *OsCIPK17*-OE, *OsCIPK17*-mutant, and WT seedlings were grown in clear water with or without 25 μmol·L^-1^. Water isolation treatment was performed on day 23 and watering was stopped, and photographs were taken and recorded at different intervals. The basic number of seedlings per pot is 30. **(B)** The survival of each strain after rehydration in **(A)** was counted, and the survival was considered when the leaves were unfolded and green. Survival rate = number of survivors/total number of seedlings. bar = 7cm. **(C)** It is the magnified field of view of *OsCIPK17*-OE surviving seedlings in **(A)**. bar = 1cm. Data in **(B)** are presented as mean ± SD of three independent experiments, and significant differences between CIPK17-OE and WT plants according to one-way ANOVA are indicated by asterisks. Asterisks indicate significance (**p < 0.01).

To investigate whether *OsCIPK17* could respond to exogenous citric acid, rice seeds were treated with 25 μmol·L^-1^ citric acid and water treatment was used as a control, and the data were photographed and observed and recorded when they grew to 14 days. The results showed that citric acid stimulated the growth of rice seedlings, and the OE plants had a more significant increase in plant height and root length (**Figure 7A**). The data showed that the height of OE plants increased by 1.3-1.4 times, while the WT and mutant increased by about 1.2 times (**Figure 7B**). Root length and plant height had similar growth trends (**Figure 7C**). To further clarify the molecular mechanism of citric acid-stimulated growth, we verified the expression of three genes associated with auxin synthesis. Indoleacetic acid-induced protein 6 (IAA6) was significantly induced to be expressed by citric acid in the root system of OE plants, 1.3-1.4 times more than WT (**Figure 7D**). Auxin-responsive GH3-like protein 5 (GH3-5) was significantly induced to be expressed by citric acid in the root system of OE plants, 2.2-2.4 times more than WT (**Figure 7E**). Indole-3-pyruvate monooxygenase (YUCCA7) was significantly induced to be expressed by citric acid in the root system of OE plants, 1.9-2.0 times more than WT, and the mutant plants showed decreased expression compared to WT, which was 0.6-0.8 fold higher (**Figure 7F**). And the drought resistance of OE plants was enhanced under exogenous citric acid treatment compared to the control. After rehydration under drought stress, some OE plants survived in the citric acid-treated group (**Figures 8A, C**), while all others died, with a survival rate of 20%-33% (**Figure 8B**). In conclusion, *OsCIPK17* responds to exogenous citric acid, which also plays an important role under drought stress and in plant growth, and its important role may be related to the auxin synthesis pathway.

### Mapping of correlation network between transcriptome and metabolome

**Figure 9:**
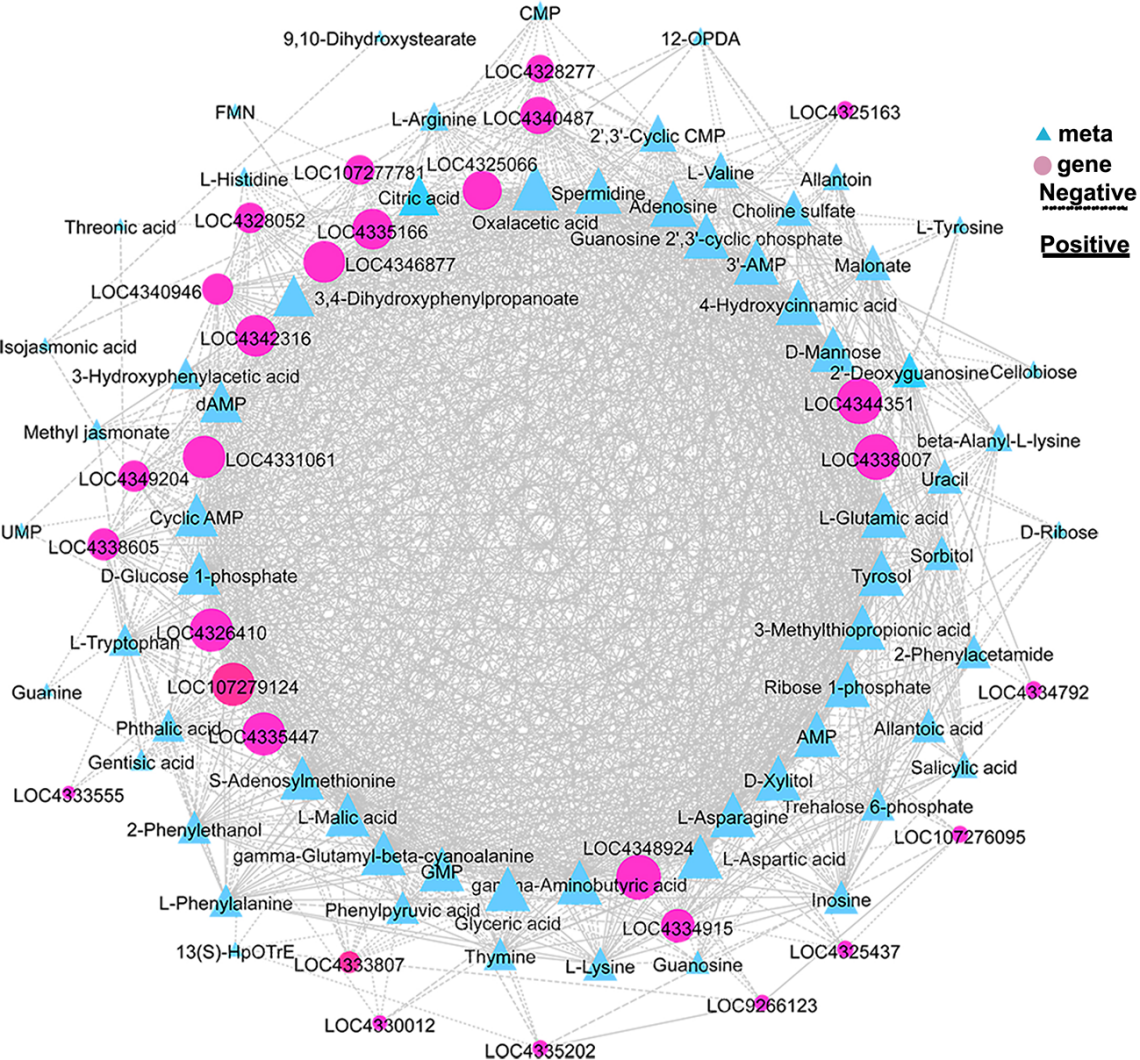
Correlation network map. Expression patterns of genes related to sugar, amino acid, nucleotide, glutathione, quinoline, and flavone metabolism and hormone signaling.

To further explore the key genes and metabolites involved in *OsCIPK17*-mediated drought response, we analyzed the correlation between genes and metabolites in the biosynthetic pathways of glucose metabolism; energy metabolism; amino acid metabolism; glutathione metabolism; plant hormone signal transduction; and metabolism of terpenoids, flavonoids, and other secondary metabolites. *OsUGT79* (*LOC4335166*), glycemic acid, l-glutamic acid, and l-aspartic acid achieved the highest scores. Among these, *OsUGT79* was strongly correlated with genes and metabolites involved in sugar metabolism, energy metabolism, plant signal transduction, metabolism of terpenoids and flavonoids, and biosynthesis of other secondary metabolites (**Figure 9**).

## Discussion

The CBL–CIPK pathway plays an important role in plant life. After binding Ca^2+^, CBL interacts with CIPK to activate it and phosphorylates the downstream target protein. However, there is no clear description of the downstream proteins of CIPK, indicating that CIPK may form a complex downstream regulatory network (Sanyal et al., 2020). *OsCIPK17* is one of the 33 *CIPK* family members in rice, although its specific functions remain unknown. In a previous study, we identified two *OsCBL*s upstream of *OsCIPK*17 and verified their interaction using Y2H and bimolecular fluorescence complementation (BiFC) assays (Gao et al., 2022a). Interestingly, *OsCBL*s and *OsCIPK17* exhibited the same phenotype under drought (**Figure S4A**). Further, the RWC (**Figure S4B**) and survival rate (**Figure S4C**) were analyzed and similar pattern to *OsCIPK17* were noted. Presumably, OsCBL8 senses the Ca^2+^ signal upon exposure to drought and activate OsCIPK17 to respond to stress (Gao et al., 2022a). Our previous research also shows that *OsCIPK17* also plays an important role in the rice filling process (Gao et al., 2022b). In the present study, OsCIPK17-OE exhibited drought tolerance through additional root growth under stress (**Figure 1D**). Root systems in plants have formed in arid areas, so that they can better absorb and use water in a water deficient environment and maintain their own growth (Kirschner et al., 2021). This suggests that deep root is a sign of plant drought resistance. Our research results show that overexpression plants have deep root characteristics under drought stress, indicating that OsCIPK17 improves drought resistance of rice by promoting root growth in physiological structure. Therefore, rice genes known to be involved in root growth under drought, including *OsDRO1* (Zhao et al., 2021), *OsNAC5* (Jeong et al., 2013) and *OsRACR5* (Kim et al., 2020) were further screened. However, Y2H assay revealed that *OsCIPK17* regulated root growth under drought without interacting with these key genes (**Figure S5B**). Moreover, ROS accumulation in *OsCIPK17*-OE plants was lower in response to drought stress. Therefore, based on previous reports, we screened OsDREB2B but found that OsCIPK17 did not interact with OsDREB2B, indicating that *OsDREB2B* did not suppress ROS accumulation in *OsCIPK17-*overexpressing plants (**Figure S5B**).

Apparently, low-molecular-weight substances play important roles in regulating cell homeostasis, and secondary metabolites are involved in interactions with the external environment (Petruk et al., 2018; Seca and Pinto, 2018). To elucidate the specific drought tolerance mechanism of *OsCIPK17*, the transcriptome and untargeted metabolome were sequenced for clarifying the regulatory mode of *OsCIPK17* on genes and metabolites. The soluble sugar content of overexpression lines was increased under drought, which was consistent with the results of our metabolomic analysis. Specifically, the content of various sugars, including erythrulose, mannose, alpha-D-glucose, and galactose, which were all reductive monosaccharides, was increased (**Figure 4**). As a major antioxidant, AsA plays a crucial role in plant growth and development (Wang et al., 2013). Upon plant exposure to adverse environments, AsA acts as an antioxidant or a cofactor in redox reactions to protect cells. The regulation of AsA synthesis is closely related to cell wall synthesis, and mannose is involved as a sugar unit in this process (Fenech et al., 2019). Therefore, CIPK17 likely regulates cell wall synthesis by altering mannose content and regulates AsA metabolism to cope with ROS accumulation for protecting plants from drought. Moreover, in the present study, KEGG entries with GO enrichment of DEGs and DEMs in the treatment groups included AsA and aldonic acid metabolism, which are the major pathways of glucose metabolism (**Figure 3E**). From these results, the increase in soluble sugars during drought is related to the alleviation of osmotic stress for maintaining intracellular homeostasis and AsA synthesis to resist cellular antioxidant responses (Fàbregas and Fernie, 2019). However, the specific regulatory mechanism warrants further exploration. Glucose metabolism belongs to the energy metabolism category (Judge and Dodd, 2020). Trehalose, a disaccharide, plays a central role in energy and metabolic homeostasis (Fernandez et al., 2010). In the present study, we detected T6P as a differential metabolite between NIP and OE9 under drought stress. T6P is used as an intermediate in trehalose synthesis. Under normal conditions, NIP and OE9 contained higher levels of T6P; however, upon drought exposure, the T6P level decreased significantly, which was due to its transformation to trehalose (**Figure 4A**). Further, other enzymes in the trehalose synthetic pathway showed similar trends (**Figure 4B**). Interestingly, the expression of trehalase, which catalyzes the conversion of trehalose to glucose, was significantly increased, indicating that the synthesized trehalose was in use. This explains the involvement of *OsCIPK17* in trehalose synthesis and metabolism to regulate energy and metabolic homeostasis and ensure plant health. The TCA cycle is an important part of plant energy metabolism. Few studies have explored the changes in the TCA cycle under drought in rice, and this study may provide some answers. Citric acid plays a key role at the initial stages of the TCA cycle. When present in excess, citric acid inhibits glycolysis, stimulates gluconeogenesis, and hinders the downstream TCA reactions. Plants assume “dormancy” to prevent cells from wasting large amounts of energy and ensure faster recovery under normal conditions (Gao et al., 2019). Consistently, OE8 and OE9 showed better recoveries in the present experiment. Light is one of the most important conditions for plant growth and development. It not only provides important energy for photosynthesis, but also plays an important role in regulating the whole process of plant growth and development as a signal factor (Choi et al., 2020). The response mechanism of plants to different light signals has been widely studied. The photoreceptors in plants, such as light pigments and cryptochrome, can perceive the changes of light signals (Wang et al., 2021). After a series of regulatory actions, the adaptability of plants will change. Light signals affect complex transcriptional regulatory networks and regulate various processes of growth and development, such as seed germination, light morphogenesis, shade avoidance response and photoperiod response. Dark stress can easily lead to leaf senescence, elongation of hypocotyls and petioles, early entry of plants into reproductive growth and other complex traits (Seluzicki et al., 2017). Yellowed rice seedlings of different genotypes by dark culture were then drought treatment (**Figure S9**), the results showed that the mutant, overexpression plant and wild type showed the same phenotype, which was different from our previous normal light conditions, in that the mutant reduced rice drought resistance and overexpression enhanced rice drought resistance (**Figure 1A**). Since there are many factors affecting plant growth under long-term dark treatment, from the perspective of plant energy, rice can only obtain energy from nutrient solution. Mutants, overexpressed lines and WT may be at the same level of energy metabolism, resulting in OsCIPK17 not participating in the phenotype of regulating drought under dark conditions. However, there are many aspects affected by dark treatment, including plant light habits and circadian rhythm. Green plants contain a lot of chloroplasts (Li and Kim, 2021), which are important organelles related to ROS clearance in plants. This needs to be explored further. It was further shown that OsCIPK17 is involved in energy metabolism pathways in response to drought. In exogenous citric acid treatment, citric acid increased the expression of auxin synthesis genes, and OsCIPK17 also responded to this process. It was shown that citric acid, auxin and OsCIPK17 have a complex regulatory relationship. Among them, the typical *DRO1* gene confers drought tolerance in rice through the auxin pathway (Zhao et al., 2021). However, whether citric acid accumulation is directly or indirectly regulated by *OsCIPK17* under drought remains unclear.

**Figure 10:**
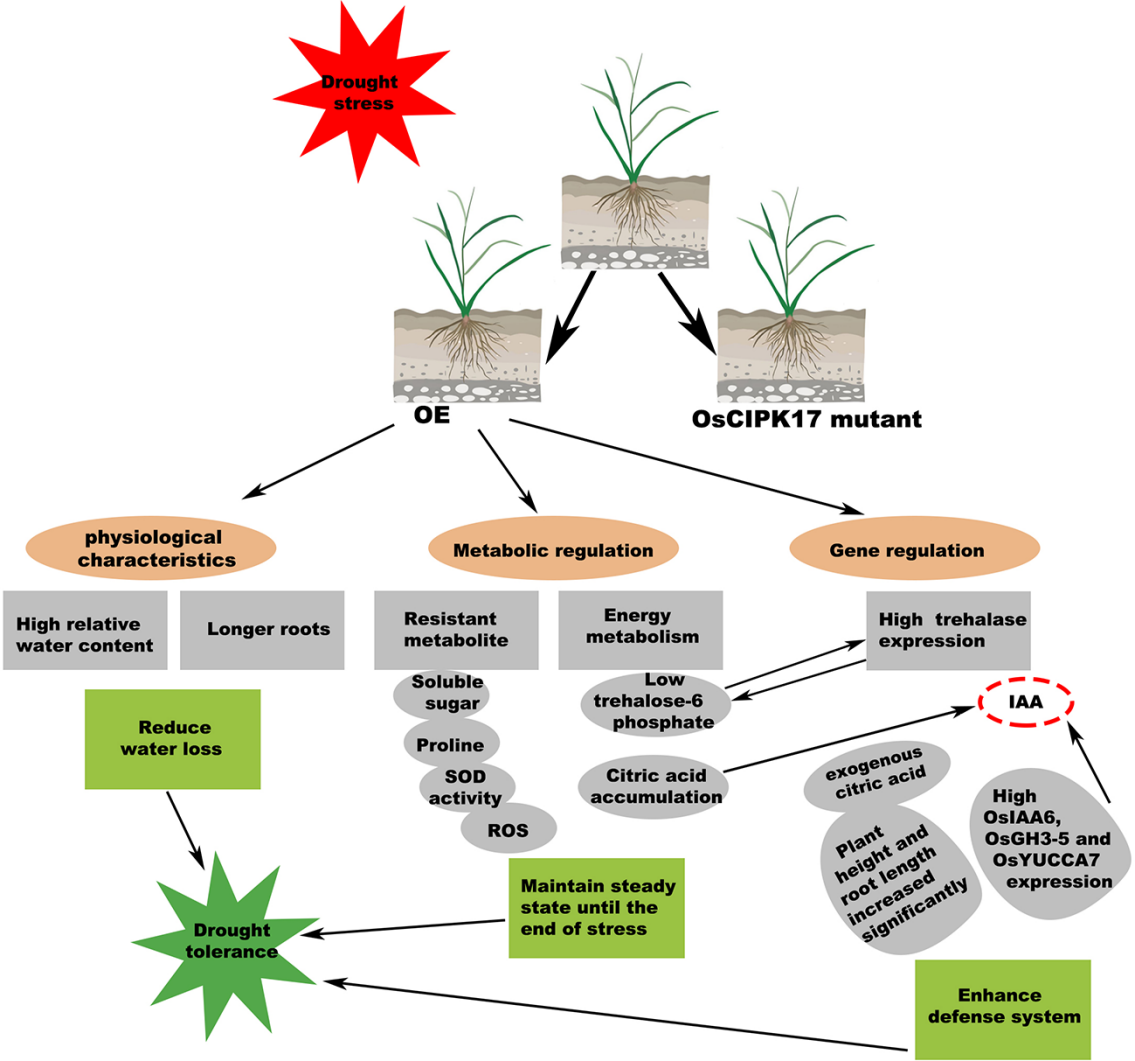
Summary of major metabolic events observed, which confer drought tolerance through *OsCIPK17*-OE in rice. The features described in this work are marked with gray ellipses. Possible connections are connected by dashed lines.

Glycosyltransferases are present in almost all organisms. They catalyze the transfer of glycosyl groups from activated donor molecules to receptor molecules, which are important biotransformation reactions. These enzymes are directly involved in the transformation of disaccharides, monoglycosides, oligosaccharides, polyglycosides, and polysaccharides (Rahimi et al., 2019). UDP-glucose is the most common glycosyl donor in plants (Gutmann et al., 2017). In addition to monosaccharides, oligosaccharides, and polysaccharides, common glycosyl receptors include non-carbohydrates, such as proteins, lipids, antibiotics, sterols, phenols, terpenes, cyanohydrins, plant hormones, alkaloids, plant toxins, and exogenous substances (Shimojima, 2011). These enzymes catalyze an array of substrates, playing vital roles in plant growth and development, metabolic regulation, detoxification of internal and external toxins, and synthesis and storage of secondary metabolites (Liu et al., 2015; Wu et al., 2019; Tezuka et al., 2021). Through transcriptional and metabolic association analysis, we found that *OsUGT79* is involved in multiple pathways. As a downstream protein of OsCIPK17 and a member of UGT family, OsUGT79 may serve multiple functions. Specifically, under drought conditions, OsCIPK17 induces root growth, which may be related to plant hormones, and OsUGT79 may catalyze plant hormone responses to complete this pathway. Our identification and phylogenetic analysis of the UGT gene family, OsUGT79, may be associated with auxin, which may imply that the increase in root length under drought conditions is caused by OsUGT79 regulating auxin. Further, OsUGT79 may be involved in the catalysis of monosaccharides and other sugars (trehalose) to regulate plant soluble sugar content and intracellular homeostasis. OsUGT79 may also regulate the TCA cycle by catalyzing the synthesis of other metabolites and protect plants by removing toxins *in vivo* and *in vitro*. Finally, through interaction with OsUGT79, OsCIPK17 may assign the regulation of intracellular homeostasis to OsUGT79 to complete various regulatory cascades and ultimately elicit drought response (**Figure 10**). Further studies on how drought is regulated at the whole gene level and at the metabolic level in rice provide new insights into the OsCIPK17-OsUGT79 regulatory network.

## Conclusions

Overexpression of *OsCIPK17* conferred greater drought tolerance in rice. combining transcriptome and non-targeted metabolome, we found that *OsCIPK17*-OE resisted drought by regulating the levels of soluble sugars, mainly reducing sugars, *in vivo*. OsCIPK17 was involved in energy regulation, including the algal sugar metabolic pathway and citric acid cycle to resist drought. More interestingly, we also demonstrated that exogenous citric acid stimulates plant growth and that auxin synthesis genes respond to OsCIPK17 to participate in it, forming a complex regulatory network to jointly resist drought.

## Data availability statement

The original contributions presented in the study are publicly available. This data can be found here: https://ngdc.cncb.ac.cn/gsa/, CRA008371.

## Author contributions

YuC and SL conceived this project and designed all experiments. SL and YaC performed the experiments. SL, YaC and BH participated in sequencing data preparation. SW, CZ, PX and YZ participated in sample collection. SL and YuC wrote the manuscript. YuC and BW revised the manuscript. All authors contributed to the article and approved the submitted version.

## Supplementary material

Supplementary Figure 1 Identification of *OsCIPK17*-OE and *OsCIPK17*- Mutant Lines. **(A)** The structural map of *OsCIPK17* gene shows the exon and intron of the gene and points out the specific mutation sites of the two mutant materials. **(B)** The relative expression of over expression *OsCIPK17* and NIP. Data in **(B)** are presented as mean ± SD of three independent experiments, and significant differences between *OsCIPK17*-OE and WT plants according to one-way ANOVA are indicated by asterisks. Asterisks indicate significance (*p < 0.05, **p < 0.01).

Supplementary Figure 2 Classification and statistics of differentially expressed genes (DEGs) and metabolites (DEMs) under normal conditions. **(A)** Volcano plot of DEGs under normal conditions (NIP-CK *vs* OE9-CK). **(B)** Volcano plot of DEMs under normal conditions (NIP-CK *vs* OE9-CK). **(C)** Total Venn diagram of all DEGs. **(D)** Total Venn diagram of all DEMs. (F) The number and proportion of 179 DEMs (NIP-D *vs* OE9-D) in various metabolites and their derivatives are listed in detail. Black boxes represent DEMs. CK: NIP or OE9 materials without drought treatment. OE9: *OsCIPK17*-OE9. D: drought treatment.

Supplementary Figure 3 KEGG analysis of differentially expressed genes (DEGs) and metabolites (DEMs). **(A)** OPLS-DA plot in the positive ion mode. **(B)** OPLS-DA plot in the negative ion mode. **(C)** KEGG analysis of DEGs showing the first 20 entries under normal conditions (NIP-CK *vs* OE9-CK). **(D)** KEGG analysis of DEMs showing the first 20 entries under normal conditions (NIP-CK *vs* OE9-CK). **(E)** KEGG analysis of DEGs showing the first 20 entries under drought conditions (NIP-CK *vs* OE9-CK). **(F)** KEGG analysis of DEMs showing the first 20 entries under drought conditions (NIP-CK *vs* OE9-CK). CK: NIP or OE9 materials without drought treatment. OE9: *OsCIPK17*-OE9.

Supplementary Figure 4 Drought tolerance of *OsCBL8*-OE plants was increased while that of *OsCBL8*-Mutant plants was decreased. **(A)** Growth phenotype of 3-week-old transgenic *OsCBL8*-OE, *OsCBL8*-Mutant, and WT plants at different stages of drought stress; bar = 7cm. **(B)** Relative water content of drought-treated plants. Parameters of water status in 3-week-old *OsCBL8*-OE, *OsCBL8*-Mutant and WT plants after drought stress treatment were measured at 10 days after drought. **(C)** Survival rates of each line after recovery was measured at 2 days after re-watering. Gao et al. (2022) have completed the identification of OsCBL8 mutant and overexpression lines (Published in the International Journal of Molecular Science on 18 October 2022).

Supplementary Figure 5 The protein yeast two-hybrid experiment that did not interact with OsCIPK17 in the current study. **(A)** genes selected from the Figure 9 a network diagram. **(B)** The key genes that have been reported to be involved in drought stress were selected in the previous work. P: positive (pGADT7-T+pGBKT7-53), N: negative (pGADT7-T+pGBKT7-lam).

Supplementary Figure 6 Phylogenetic tree of the UGT gene family members in rice. The phylogenetic tree was divided into five branches based on evolutionary relationships, in which the purple color was associated with flavonoid synthesis, the red part of the gene function was not clarified, the brown color was associated with anthocyanin synthesis, the blue color was associated with growth hormone synthesis and the burgundy color was associated with cytokinin synthesis.

Supplementary Figure 7 Correlation test of gene expression level among samples. Pearson correlation coefficient is used to express the gene expression level correlation among samples in the heat map grid.

Supplementary Figure 8 Metabolic group quality control and quality assurance. **(A)** QC detection in positive ion mode. **(B)** QC detection in negative ion mode. **(C)** QA detection in positive ion mode. **(D)** QA detection in negative ion mode. The reliability is reflected in the PCA analysis chart. The red dots represent QC samples, and the other color dots are samples. If QC samples gather, it indicates that the repeatability is good.

Supplementary Figure 9 *OsCIPK17*-OE, *OsCIPK17* Mutant and WT plants showed phenotypes under drought treatment. Growth phenotype of 3-week-old transgenic *OsCIPK17*-OE, *OsCIPK17*-Mutant, and WT plants at different stages of drought stress; bar = 7cm. The rice seedlings at the age of 3 weeks were treated by cutting off water. All experiments were conducted in dark conditions. The drought phenotype was photographed 2 weeks later, followed by watering, and the photos were taken 2 days later. Experiments were repeated three times, and similar results were obtained.

Supplementary Table 1 Specific primer sequence
