# Supplementary figures and images for: Combined metabolomic and transcriptomic analysis reveals key components of OsCIPK17 overexpression improves drought tolerance in rice
Source: Front Plant Sci. 2023 Jan 9;13:1043757. doi: 10.3389/fpls.2022.1043757 (PMC9868928; doi:10.3389/fpls.2022.1043757)

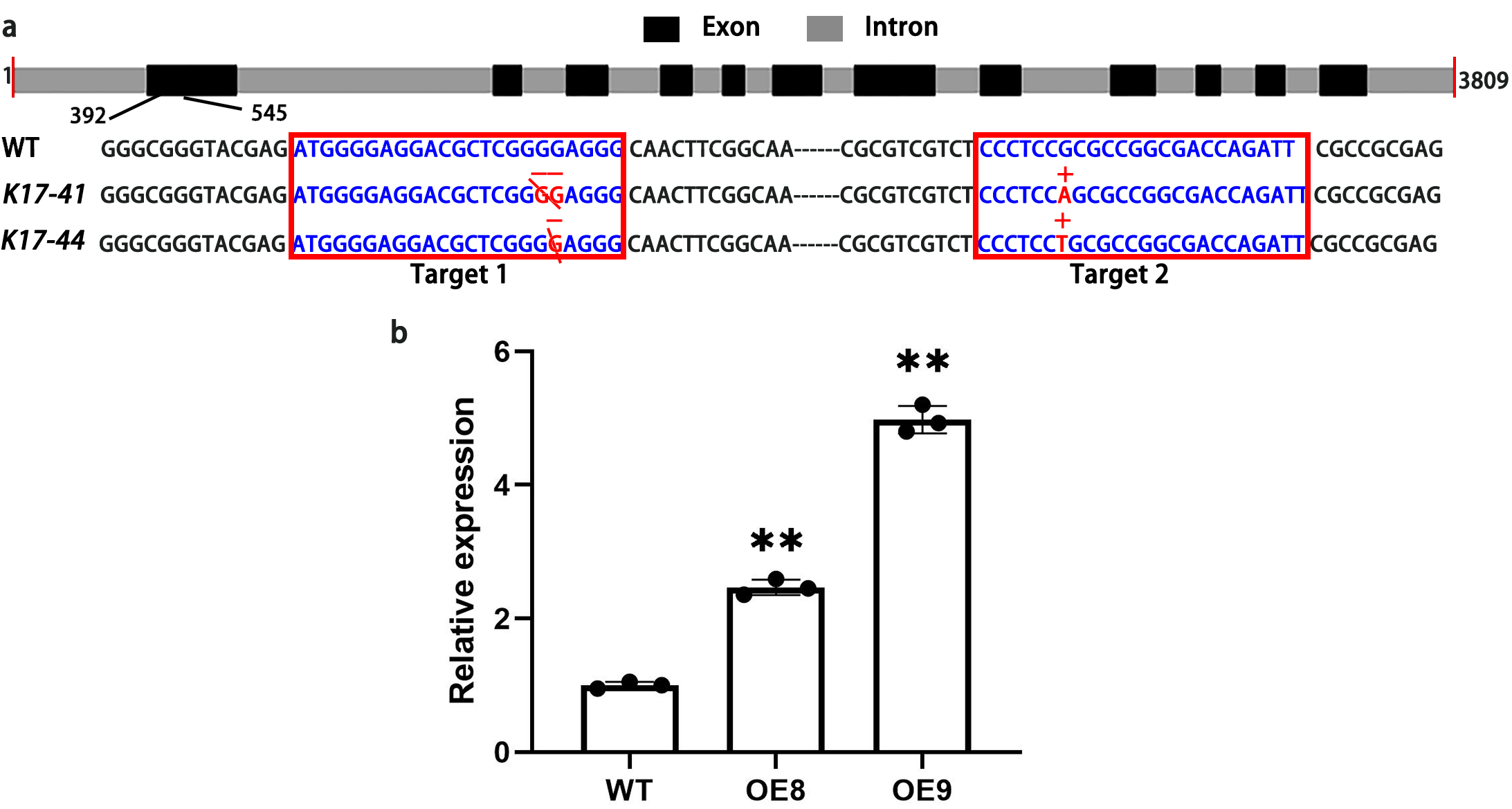

Supplement: Supplementary Figure 1 — Identification of OsCIPK17-OE and OsCIPK17- Mutant Lines. (A) The structural map of OsCIPK17 gene shows the exon and intron of the gene and points out the specific mutation sites of the two mutant materials. (B) The relative expression of over expression OsCIPK17 and NIP. Data in (B) are presented as mean ± SD of three independent experiments, and significant differences between OsCIPK17-OE and WT plants according to one-way ANOVA are indicated by asterisks. Asterisks indicate significance (*p < 0.05, **p < 0.01). [file Image_1.jpeg]

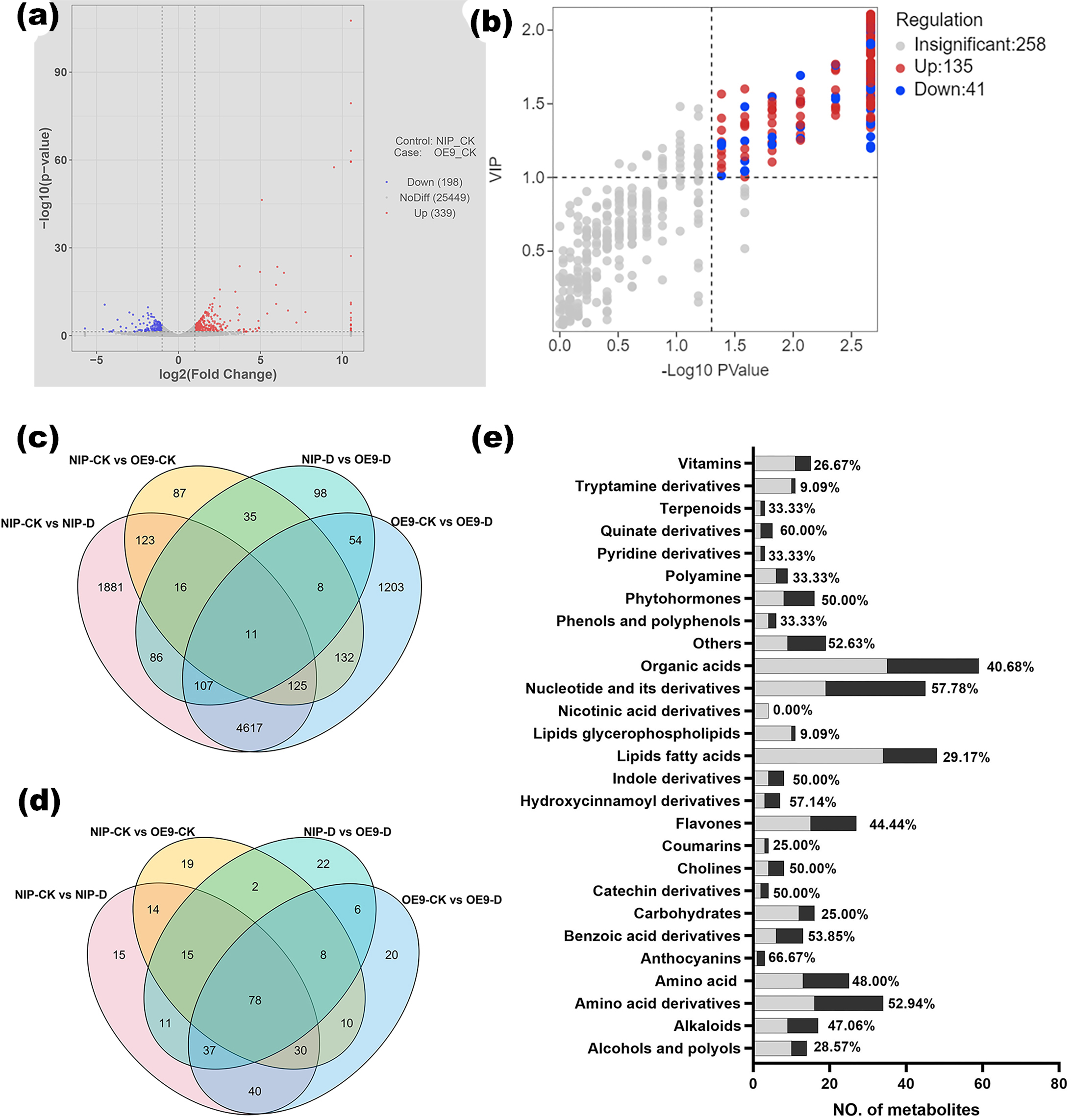

Supplement: Supplementary Figure 2 — Classification and statistics of differentially expressed genes (DEGs) and metabolites (DEMs) under normal conditions. (A) Volcano plot of DEGs under normal conditions (NIP-CK vs OE9-CK). (B) Volcano plot of DEMs under normal conditions (NIP-CK vs OE9-CK). (C) Total Venn diagram of all DEGs. (D) Total Venn diagram of all DEMs. (F) The number and proportion of 179 DEMs (NIP-D vs OE9-D) in various metabolites and their derivatives are listed in detail. Black boxes represent DEMs. CK: NIP or OE9 materials without drought treatment. OE9: OsCIPK17-OE9. D: drought treatment. [file Image_2.jpeg]

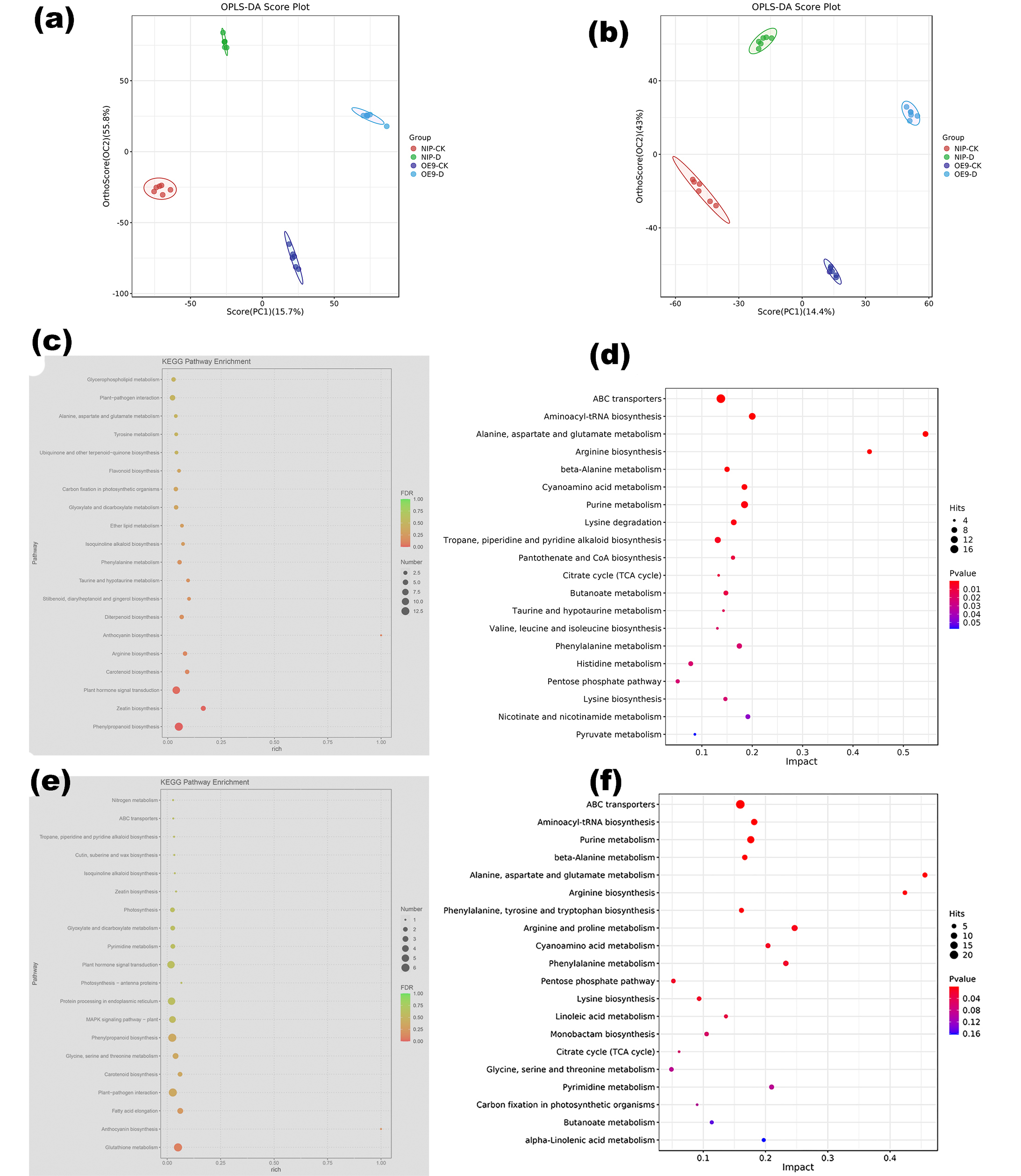

Supplement: Supplementary Figure 3 — KEGG analysis of differentially expressed genes (DEGs) and metabolites (DEMs). (A) OPLS-DA plot in the positive ion mode. (B) OPLS-DA plot in the negative ion mode. (C) KEGG analysis of DEGs showing the first 20 entries under normal conditions (NIP-CK vs OE9-CK). (D) KEGG analysis of DEMs showing the first 20 entries under normal conditions (NIP-CK vs OE9-CK). (E) KEGG analysis of DEGs showing the first 20 entries under drought conditions (NIP-CK vs OE9-CK). (F) KEGG analysis of DEMs showing the first 20 entries under drought conditions (NIP-CK vs OE9-CK). CK: NIP or OE9 materials without drought treatment. OE9: OsCIPK17-OE9. [file Image_3.jpeg]

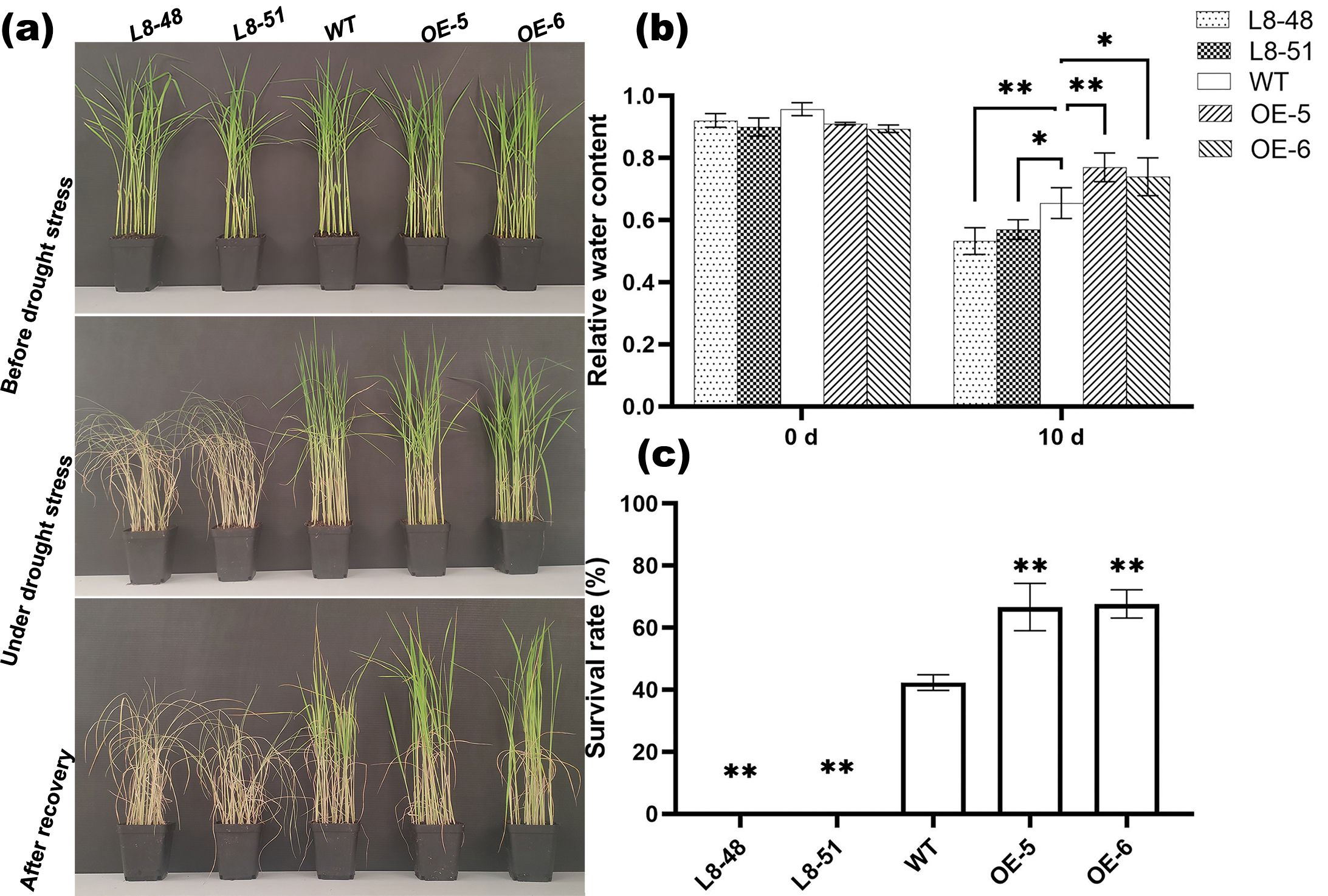

Supplement: Supplementary Figure 4 — Drought tolerance of OsCBL8-OE plants was increased while that of OsCBL8-Mutant plants was decreased. (A) Growth phenotype of 3-week-old transgenic OsCBL8-OE, OsCBL8-Mutant, and WT plants at different stages of drought stress; bar = 7cm. (B) Relative water content of drought-treated plants. Parameters of water status in 3-week-old OsCBL8-OE, OsCBL8-Mutant and WT plants after drought stress treatment were measured at 10 days after drought. (C) Survival rates of each line after recovery was measured at 2 days after re-watering. Gao et al. (2022) have completed the identification of OsCBL8 mutant and overexpression lines (Published in the International Journal of Molecular Science on 18 October 2022). [file Image_4.jpeg]

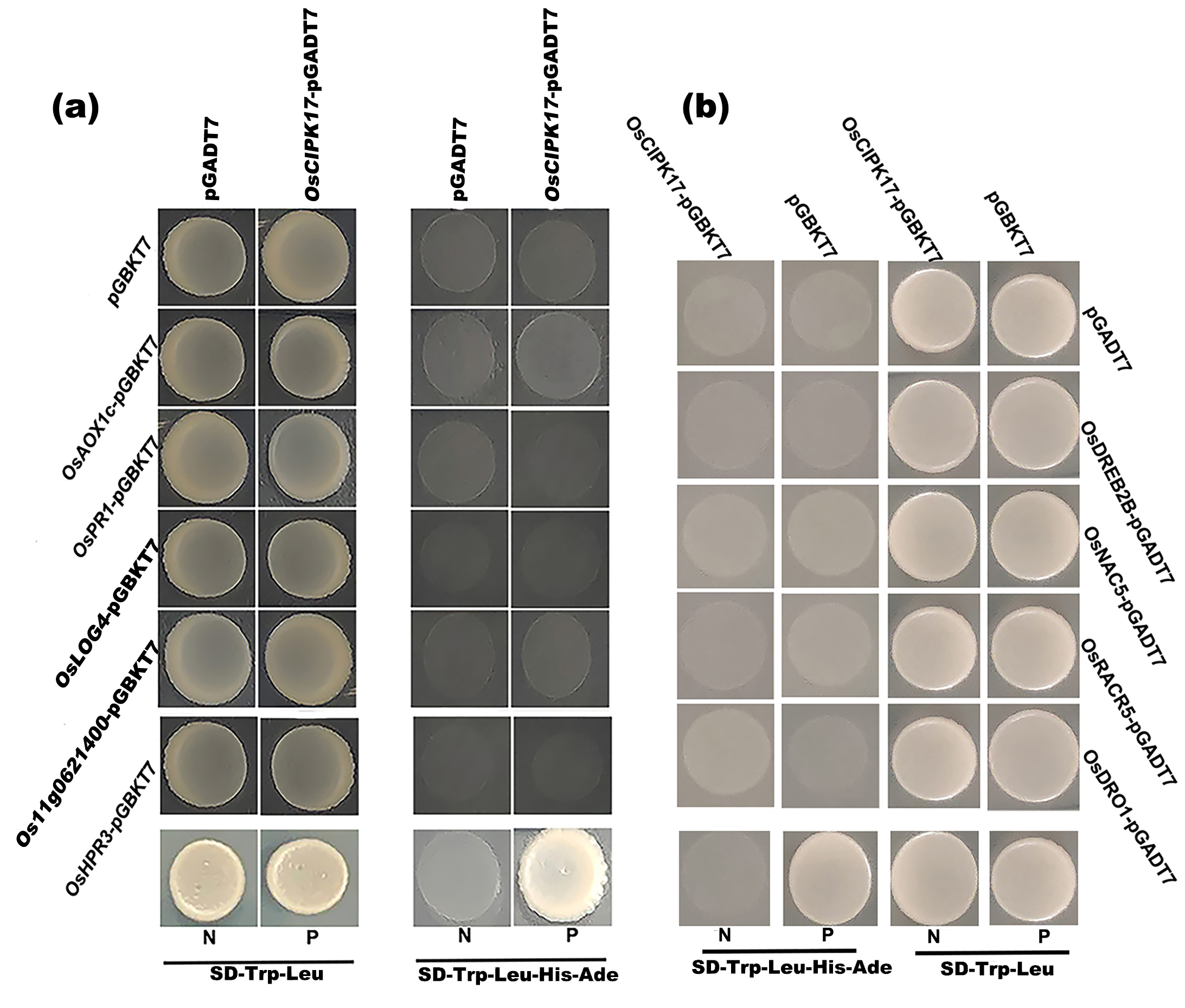

Supplement: Supplementary Figure 5 — The protein yeast two-hybrid experiment that did not interact with OsCIPK17 in the current study. (A) genes selected from the Figure 9 a network diagram. (B) The key genes that have been reported to be involved in drought stress were selected in the previous work. P: positive (pGADT7-T+pGBKT7-53), N: negative (pGADT7-T+pGBKT7-lam). [file Image_5.jpeg]

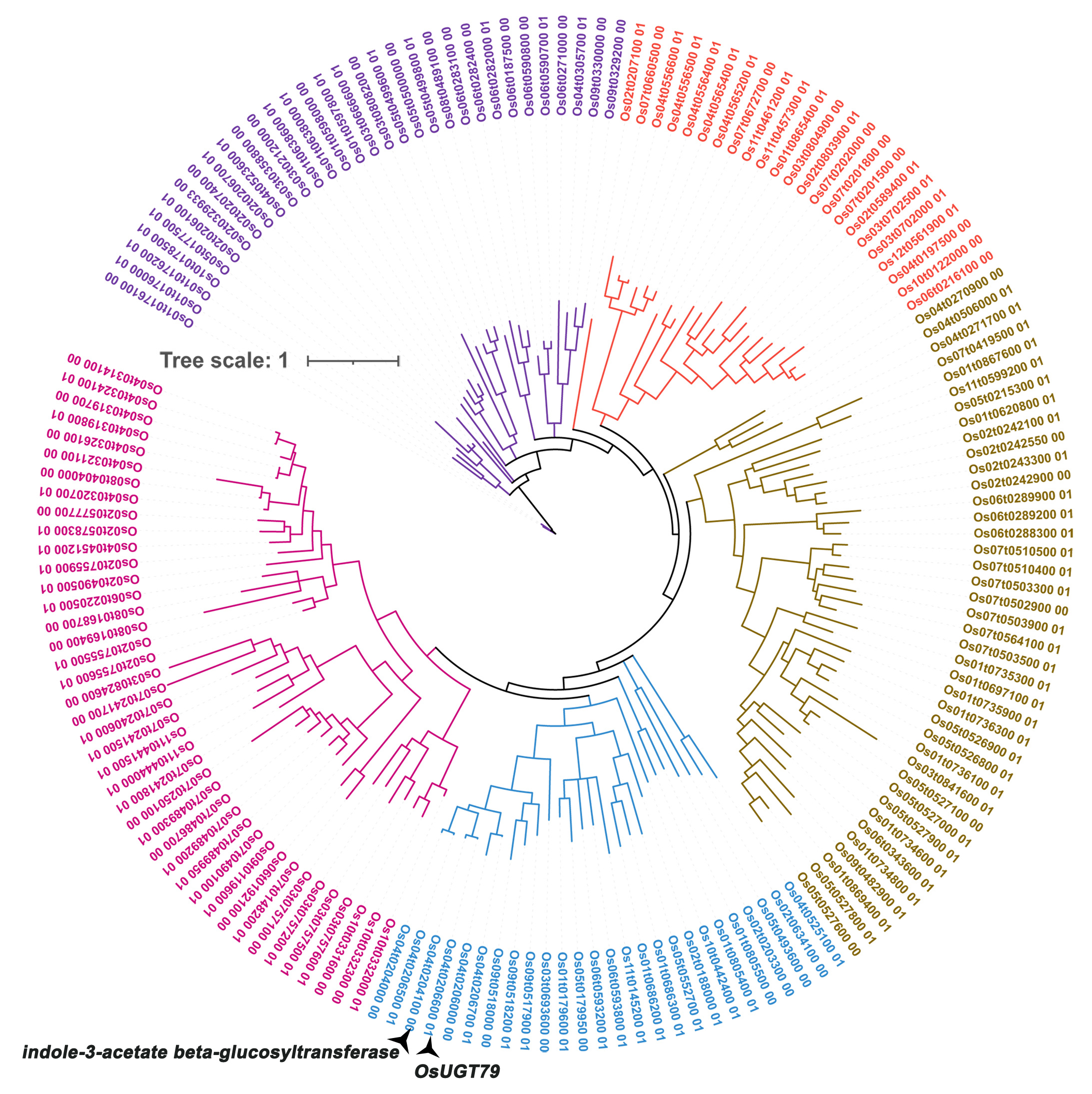

Supplement: Supplementary Figure 6 — Phylogenetic tree of the UGT gene family members in rice. The phylogenetic tree was divided into five branches based on evolutionary relationships, in which the purple color was associated with flavonoid synthesis, the red part of the gene function was not clarified, the brown color was associated with anthocyanin synthesis, the blue color was associated with growth hormone synthesis and the burgundy color was associated with cytokinin synthesis. [file Image_6.jpeg]

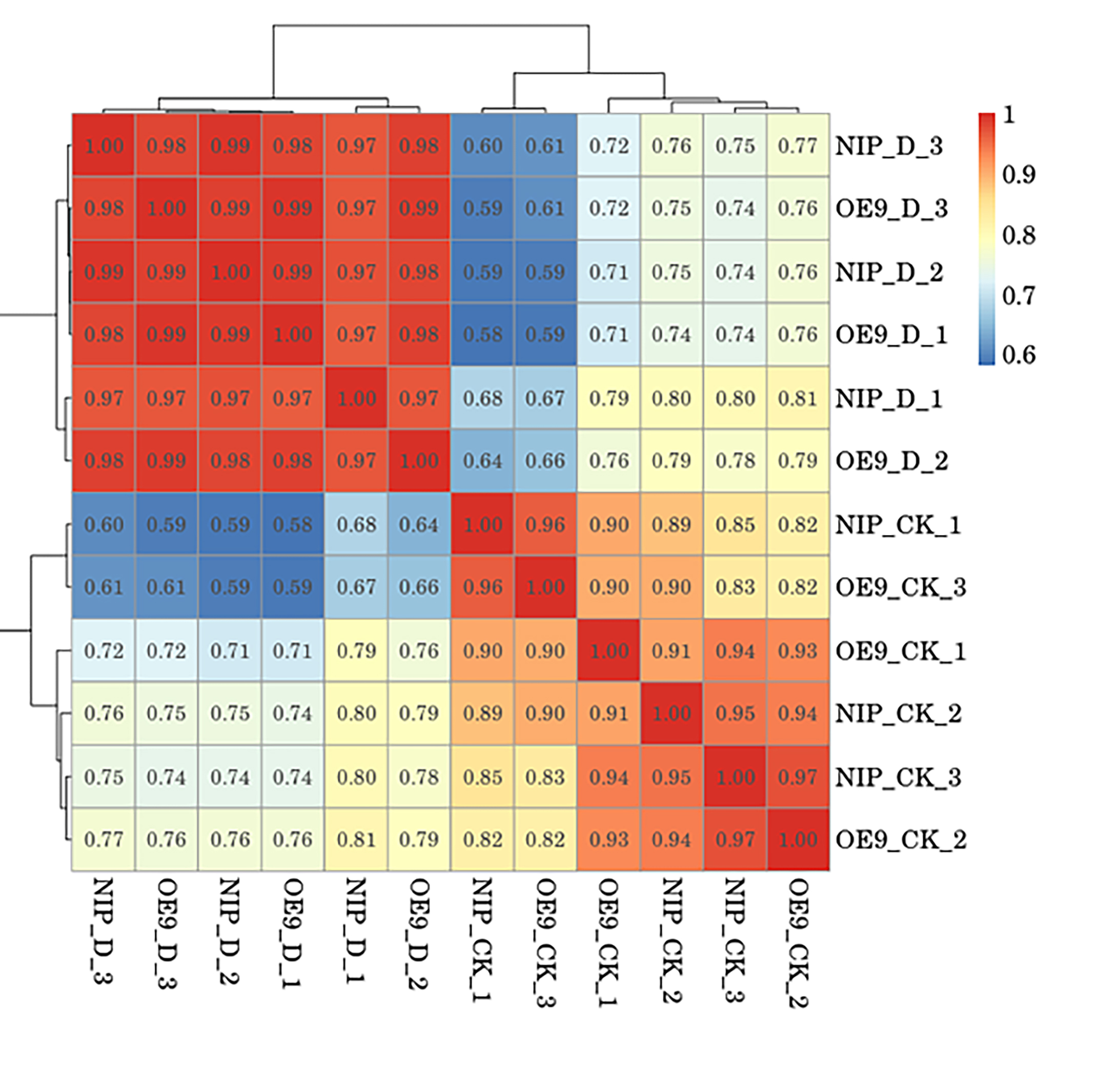

Supplement: Supplementary Figure 7 — Correlation test of gene expression level among samples. Pearson correlation coefficient is used to express the gene expression level correlation among samples in the heat map grid. [file Image_7.jpeg]

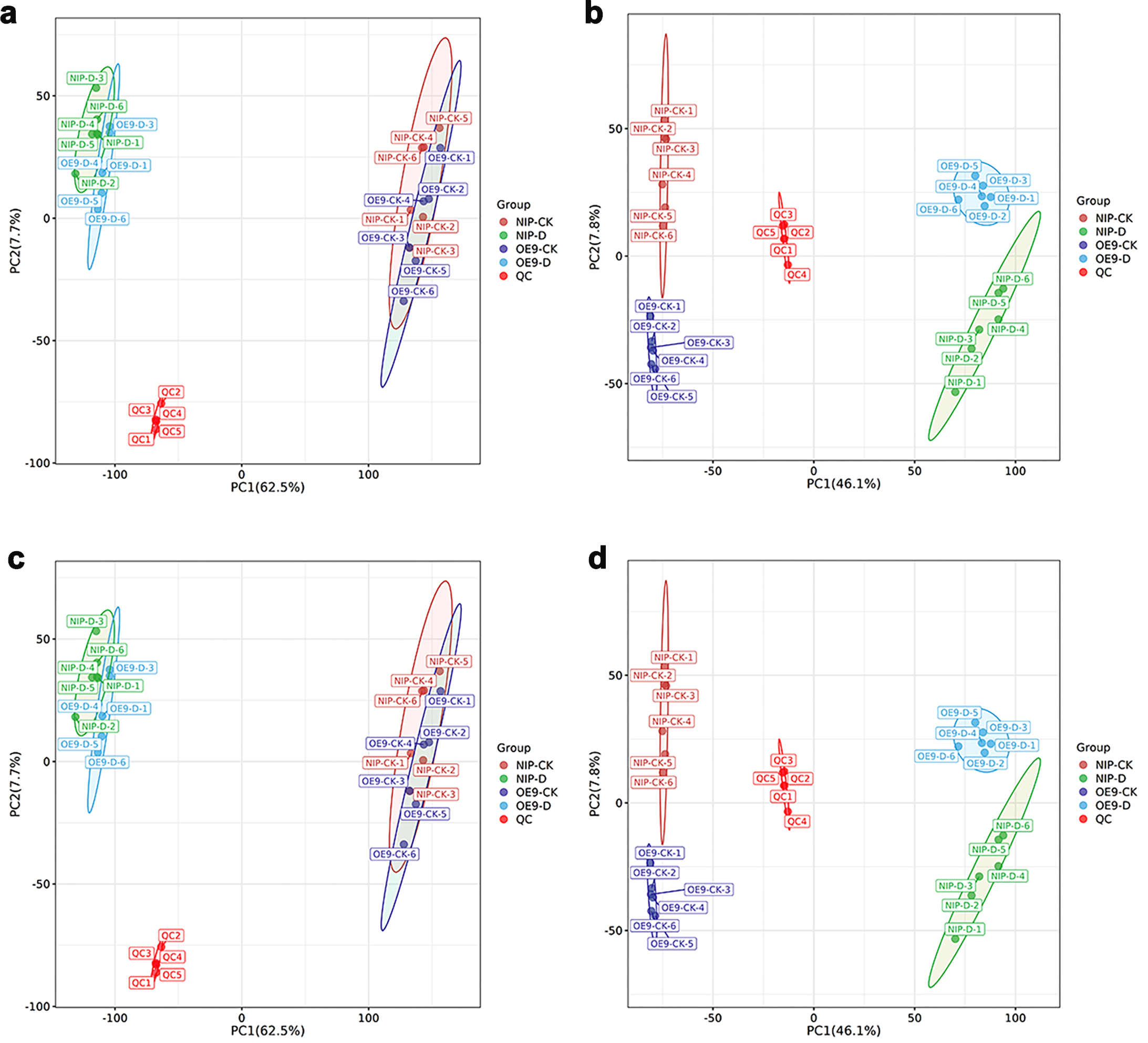

Supplement: Supplementary Figure 8 — Metabolic group quality control and quality assurance. (A) QC detection in positive ion mode. (B) QC detection in negative ion mode. (C) QA detection in positive ion mode. (D) QA detection in negative ion mode. The reliability is reflected in the PCA analysis chart. The red dots represent QC samples, and the other color dots are samples. If QC samples gather, it indicates that the repeatability is good. [file Image_8.jpeg]

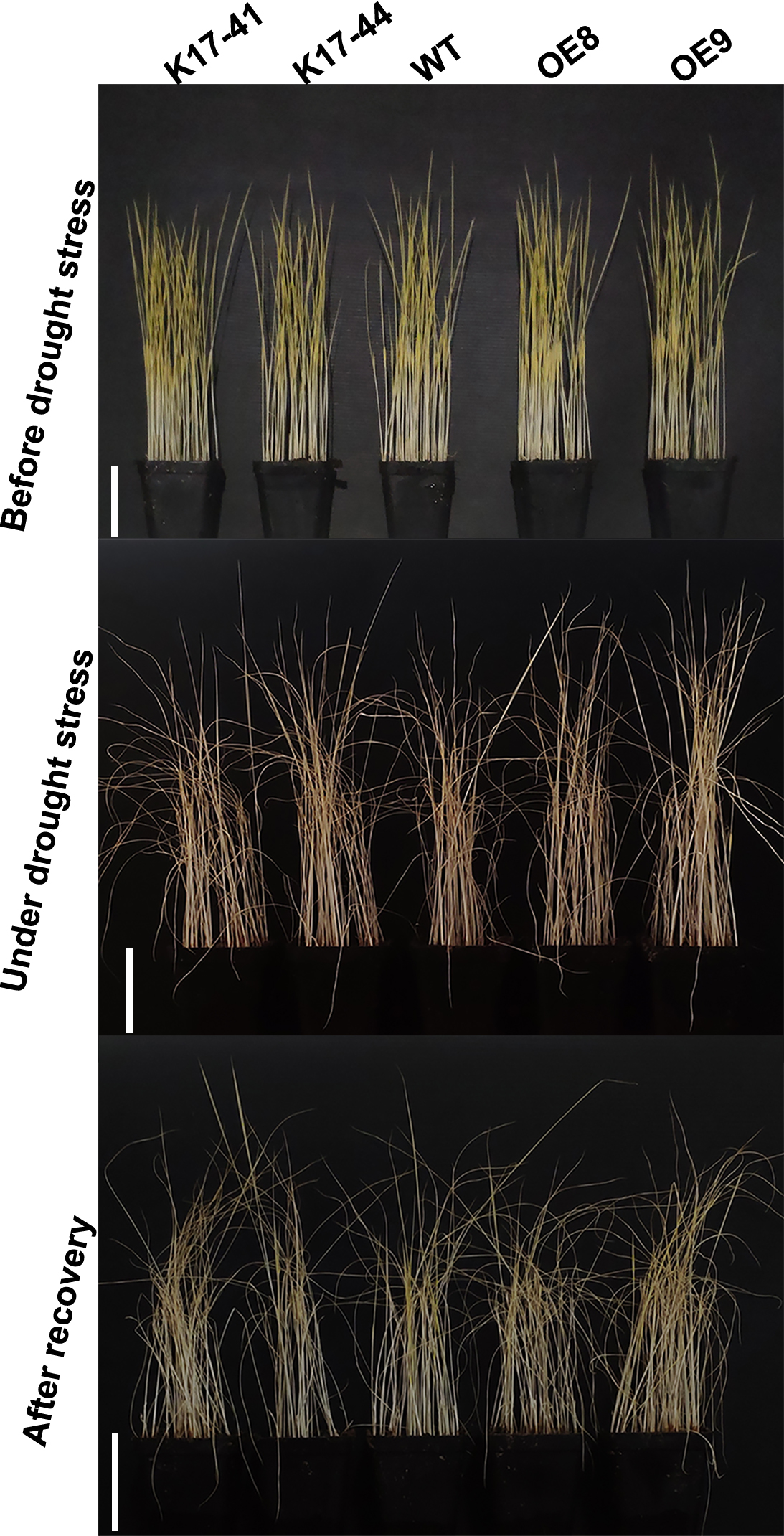

Supplement: Supplementary Figure 9 — OsCIPK17-OE, OsCIPK17 Mutant and WT plants showed phenotypes under drought treatment. Growth phenotype of 3-week-old transgenic OsCIPK17-OE, OsCIPK17-Mutant, and WT plants at different stages of drought stress; bar = 7cm. The rice seedlings at the age of 3 weeks were treated by cutting off water. All experiments were conducted in dark conditions. The drought phenotype was photographed 2 weeks later, followed by watering, and the photos were taken 2 days later. Experiments were repeated three times, and similar results were obtained. [file Image_9.jpeg]
